# Delivering integrated strategies from a mobile unit to address the intertwining epidemics of HIV and addiction in people who inject drugs: the HPTN 094 randomized controlled trial protocol (the INTEGRA Study)

**DOI:** 10.1186/s13063-023-07899-5

**Published:** 2024-02-15

**Authors:** David Goodman-Meza, Steven Shoptaw, Brett Hanscom, Laramie R. Smith, Philip Andrew, Irene Kuo, Jordan E. Lake, David Metzger, Ellen A. B. Morrison, Melissa Cummings, Jessica M. Fogel, Paul Richardson, Jayla Harris, Jesse Heitner, Sarah Stansfield, Nabila El-Bassel, for the HPTN 094 Study Team

**Affiliations:** 1 Division of Infectious Diseases, David Geffen School of Medicine, University of California, Los Angeles, 10833 Le Conte Ave., CHS 52-215, Los Angeles, CA 90095-1688 USA; 2 Department of Family Medicine, University of California, Los Angeles, Los Angeles, CA USA; 3 Statistical Center for HIV/AIDS Research and Prevention (SCHARP), Seattle, WA USA; 4 Division of Infectious Diseases and Global Public Health, University of California, San Diego, San Diego, CA USA; 5 Family Health International (FHI 360), Durham, NC USA; 6 Milken Institute School of Public Health Department of Epidemiology, George Washington University, Washington, DC, USA; 7 UTHealth-Houston, Houston, TX USA; 8 Department of Psychiatry, Perelman School of Medicine, University of Pennsylvania, Philadelphia, PA USA; 9 ICAP, Mailman School of Public Health, Columbia University, New York, NY USA; 10 Statistical Center for HIV/AIDS Research and Prevention, Fred Hutchinson Cancer Center, Seattle, WA USA; 11 Department of Pathology, Johns Hopkins University School of Medicine, Baltimore, MD USA; 12 Division of Infectious Diseases, Massachusetts General Hospital, Boston, MA USA; 13 Vaccine and Infectious Disease Division, Fred Hutchinson Cancer Center, Seattle, WA USA; 14 School of Social Work, Columbia University, New York, NY USA

## Abstract

**Background:**

Persons with opioid use disorders who inject drugs (PWID) in the United States (US) face multiple and intertwining health risks. These include interference with consistent access, linkage, and retention to health care including medication for opioid use disorder (MOUD), HIV prevention using pre-exposure prophylaxis (PrEP), and testing and treatment for sexually transmitted infections (STIs). Most services, when available, including those that address substance misuse, HIV prevention, and STIs, are often provided in multiple locations that may be difficult to access, which further challenges sustained health for PWID. HPTN 094 (INTEGRA) is a study designed to test the efficacy of an integrated, “whole-person” strategy that provides integrated HIV prevention including antiretroviral therapy (ART), PrEP, MOUD, and STI testing and treatment from a mobile health delivery unit (“mobile unit”) with peer navigation compared to peer navigation alone to access these services at brick and mortar locations.

**Methods:**

HPTN 094 (INTEGRA) is a two-arm, randomized controlled trial in 5 US cities where approximately 400 PWID without HIV are assigned either to an experimental condition that delivers 26 weeks of “one-stop” integrated health services combined with peer navigation and delivered in a mobile unit or to an active control condition using peer navigation only for 26 weeks to the same set of services delivered in community settings. The primary outcomes include being alive and retained in MOUD and PrEP at 26 weeks post-randomization. Secondary outcomes measure the durability of intervention effects at 52 weeks following randomization.

**Discussion:**

This trial responds to a need for evidence on using a “whole-person” strategy for delivering integrated HIV prevention and substance use treatment, while testing the use of a mobile unit that meets out-of-treatment PWID wherever they might be and links them to care systems and/or harm reduction services. Findings will be important in guiding policy for engaging PWID in HIV prevention or care, substance use treatment, and STI testing and treatment by addressing the intertwined epidemics of addiction and HIV among those who have many physical and geographic barriers to access care.

**Trial registration:**

ClinicalTrials.gov NCT04804072. Registered on 18 March 2021.

**Supplementary Information:**

The online version contains supplementary material available at 10.1186/s13063-023-07899-5.

## Contributions to the literature

- HPTN 094 (INTEGRA) is a randomized controlled trial evaluating the use of mobile health units with peer navigation in providing integrated prevention and care to engage and improve health outcomes in people who inject drugs (PWID) versus peer navigation to community services alone.
- HPTN 094 can serve as a model to implement integrated, “whole-person” care including medications for opioid use disorder and HIV prevention and a limited set of primary care and harm reduction services provided from a mobile unit with linkage to care using peer navigation.
- The study includes an implementation science evaluation leveraging mixed qualitative and quantitative methods to bring knowledge to practice and to understand barriers and facilitators of implementing the delivery of integrated HIV, substance and infectious diseases treatments in mobile units, and linkage to care using peer navigation.
- The study is being conducted in five US cities that have differing cultural, geographic, and drug use landscapes that impact substance use treatment and HIV prevention for PWID which are captured through the implementation science protocols.

## Introduction

Fatal drug overdoses continue to climb in the United States (US), with more than 107,000 deaths reported in 2021 [1]. Of concern, the rate of increase in overdose deaths is disproportionately high for Latinx and Black/African Americans [2]. In the current “fourth wave” of the US overdose crisis, synthetic opioids such as fentanyl are flooding the drug supply in most jurisdictions and are now being consumed with psychostimulants (methamphetamine, cocaine) [3]. The overdose crisis continues despite relaxation in requirements for providers to prescribe buprenorphine for opioid use disorder (OUD) [4] and to increased access to methadone take-home dispensing [5], both adjustments resulting from the COVID-19 pandemic. To date, however, only 15% of those with OUD in the US are receiving medications for opioid use disorder (MOUD) [6–9], with significantly fewer individuals who identify as racial and ethnic minorities with OUD receiving MOUD [10].

Health risks for persons with OUD who inject drugs in the US include multiple and overlapping problems that interfere with consistent access to health care, particularly when the substance use disorder is active [11]. As such, risks for health threats, such as infectious diseases, continue largely unaddressed. In communities of people who inject drugs (PWID), blood-borne infections are efficiently transmitted when injection equipment and paraphernalia are shared [12]. Among people with OUD, physical and psychological discomfort from opioid withdrawal can motivate the sharing of drugs or injection equipment. Receptive syringe sharing was reported by 32% of PWID in the CDC National HIV Behavioral Surveillance (NHBS) System, and sharing other injection equipment was reported by 48% of PWID surveyed [13]. Indeed, injection drug use is the primary driver for doubling hepatitis C virus (HCV) incidence in the US, with the burden for HCV outbreaks being closely co-located with areas of the opioid crisis, particularly among PWID [14]. Unsafe injection practices that drive HCV incidence often foreshadow increases in HIV incidence; most PWID living with HIV are co-infected with HCV [15]. Of concern, among PWID, new diagnoses of HIV have reversed their decline from the start of the HIV epidemic through the 2010s and are increasing once again [16]. More recently, injection drug use has been associated with multiple HIV outbreaks across the US, Europe, and Israel [17, 18].

Behaviorally disorganizing effects of opioid and other drug use disorders interfere with the ability to plan and execute tasks such as travel, qualification for healthcare entitlements, and housing and may support ongoing drug use in lieu of seeking healthcare. Judgmental attitudes from policymakers and health care providers (stigma) further alienate persons with addiction from care-seeking. Persons with active OUD, including poly-substance use, often spend time in shared spaces, such as encampments for people experiencing homelessness, transitional housing settings, syringe services programs, parks, and tourist areas, among others.

These problems call for an HIV prevention response that meets people where they are geographically, with the aim to reduce barriers at each step in the process of accessing and sustaining MOUD, HIV prevention including pre-exposure prophylaxis (PrEP), and care for co-occurring health problems such as sexually transmitted infections (STI), HCV, and mental health. Moreover, there is a need to address barriers that interfere with entry and retention in MOUD and with sustaining relevant HIV-related outcomes, as lowering barriers to access MOUD improves HIV care outcomes and prevents death among PWID with HIV [19]. Among PWID who face stigma, discrimination, and health disparities related to the range of identities held by these individuals (e.g., as people who provide transactional sex, men who have sex with men (MSM), racial/ethnic and sexual/gender minorities, and people who use multiple substances), lowering barriers requires a strategy that moves beyond available resources in existing brick-and-mortar settings [11]. The mobile medical unit offers the potential for delivering services that are valued to persons where they are, with services being delivered without stigma and with cultural competence [20].

This study uses a randomized design to evaluate a mobile unit-delivered health intervention in addressing the overlapping health challenges to engaging and retaining PWID in HIV treatment and prevention services at a time when OUD has re-emerged as a driver of HIV infections in the US. Disruptions in HIV treatment and prevention in the setting of untreated OUD in persons who often experience co-occurring mental health and substance use disorders and negative social determinants of health contributed to multiple HIV-outbreaks in Indiana, Massachusetts, Washington, and West Virginia [21–23]. Indeed, new cases of HIV among PWID shifted from annual decreases between 2010 and 2014 [16, 24] to recent annual increases [25]. One potential reason is low PrEP uptake in PWID. In the US, PrEP uptake is estimated to be only about 26% among those with PrEP indications through June 2022 [26], yet uptake in PWID is negligible at around 1% or less [13, 27]. In response, this protocol design integrates engagement and retention for HIV prevention with the parallel effort to engage and retain PWID with OUD in treatment using MOUD (i.e., buprenorphine, methadone) [28, 29]. This approach emphasizes MOUD as a primary outcome and underscores its potential to increase uptake and adherence to PrEP or interventions for co-occurring health threats.

This paper details the rationale and structure of a study that empirically tests the relative benefits of delivering integrated health services within a mobile unit setting using peer navigation. The study is designed to evaluate the efficacy and the impact of providing integrated care services, including the provision of MOUD treatment, PrEP (or ART), STI testing and treatment, primary health care, HCV testing and referral to treatment, and harm reduction from a mobile health delivery unit (“mobile unit”), combined with peer navigation, compared with an active control arm that receives peer navigation to similar health services available at community-based agencies.

## Methods

### Overview

The study is being conducted through the HIV Prevention Trials Network (HPTN) and its leadership and operations center, FHI 360. The HPTN infrastructure is funded by and operates under the auspices of the National Institute of Allergy and Infectious Diseases (NIAID) at the US National Institutes of Health (NIH). NIAID is the sponsor of the trial and provides technical assistance, advice, and coordination of the study and carries out review and regulatory functions as well as contracting independent monitors. The study is funded through the National Institute on Drug Abuse (NIDA). Gilead Sciences donated PrEP and ART medications (Truvada, Descovy, and Biktarvy) for the trial. NIDA’s Scientific Officer advised protocol development. Gilead Sciences had no role in the study design, data collection, or analysis. We followed the Standardized Protocol Items: Recommendations for Interventional Trials (SPIRIT) reporting guidelines reporting during the drafting of this article (see Supplementary Appendix 3 for checklist).

### Study design

**Fig. 1:**
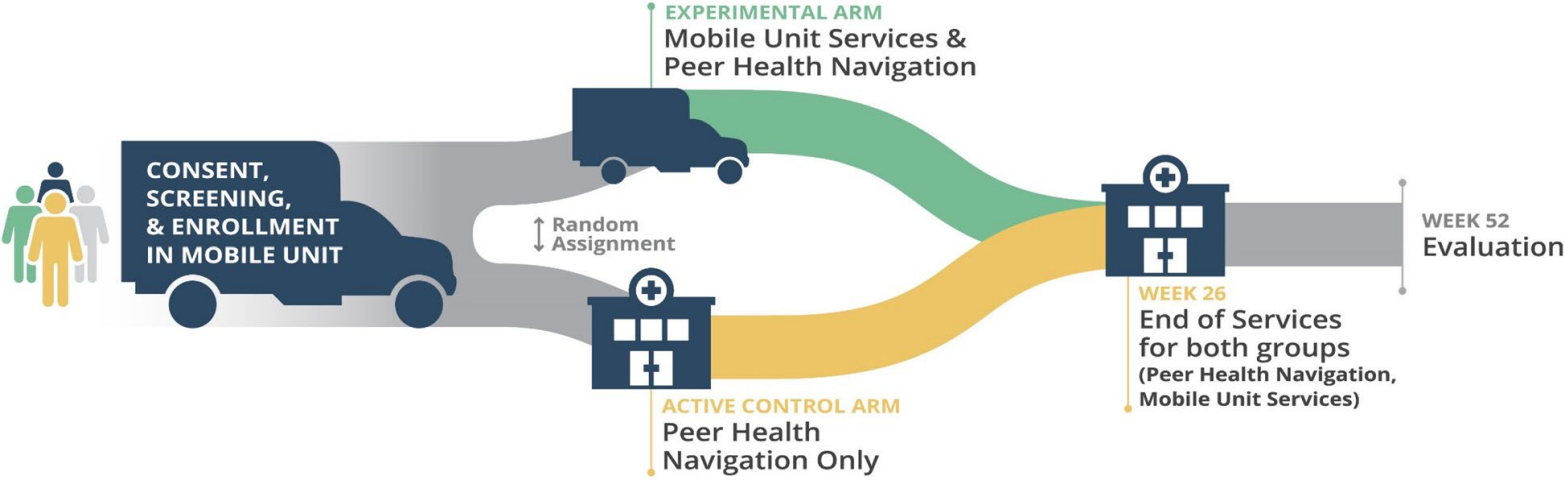
Study schema

HPTN 094 is an ongoing two-arm, controlled, individually randomized, open-label study conducted in five major US cities. The study tests whether participants randomly assigned to an experimental condition providing 26 weeks of “one-stop” integrated health services [30] delivered in a mobile unit, supported by peer navigation, improve retention on MOUD and increase retention on PrEP as measured at 26-week visits, when compared to an active control condition providing 26 weeks of peer navigation to similar health services in community agencies. By using peer navigation in both study arms, the design isolates the impact of the delivery of integrated services using a mobile medical unit. The 26-week horizon of the provision of services is intended to test the mobile units as bridges to community care. Ethical issues related to the design involved the need to create an “active control” condition for the study instead of relying on “treatment as usual,” as only limited and uncoordinated services exist for PWID not engaged in care in the US. The intensity and type of peer-navigation for both conditions are intended to be equivalent, further isolating the measurement of integrated care delivery in a mobile medical unit (intervention) compared to potentially fragmented service provision in community settings. The study schema is presented in Fig. 1.

Secondary objectives test the effects of the mobile intervention along outcomes including (a) use of MOUD at 52-week visits, (b) use of PrEP at 52-week visits, (c) opioid and polysubstance use at 26- and 52-week visits, (d) prevalence of bacterial STIs at 26- and 52-week visits, (e) fatal and non-fatal overdose events prior to 26- and 52-week visits, (f) undetectable HCV RNA at 26- and 52-week visits (among those with chronic HCV at enrollment), and (g) HCV incidence at 52-week visits (for those who are HCV negative at enrollment).

Embedded within the protocol for HPTN 094, an implementation science evaluation was initiated prior to study initiation and continued throughout the conduct of the study. The implementation science evaluation features mixed methods procedures that describe the local and cross-site contextual facilitators and barriers of the implementation of the study and to inform data-driven decisions about where to locate the mobile unit in local communities (see below for full description).

### Study sites selection

Potential HPTN clinical research sites completed an in-depth survey to evaluate staffing resources, relevant population living with addiction and infectious diseases, and prior research experience with PWID and HIV prevention and treatment. Sites were chosen by a selection committee, with selections approved by HPTN leadership, based on historical capacity to recruit PWID and people with HIV (PWH) and without HIV (PWOH), site’s experience with using MOUD and HIV medications, prior research experience, and lack of competing research commitments. The sites selected for the HPTN 094 study were the Bronx Prevention Center of ICAP at Columbia University (New York, NY), University of Pennsylvania (Philadelphia, PA), George Washington University (Washington, DC), UTHealth (Houston, TX), and University of California, Los Angeles (Los Angeles, CA).

### Sample size

HPTN 094 was designed to be serostatus neutral regarding the measurement of intervention effects on ART and PrEP utilization for PWH and PWOH, respectively. The study was originally designed to enroll similar numbers of PWH (*n* = 460) and PWOH (*n* = 400). A key finding after activating the study was that the frequency of enrollment of PWH who also inject opioids was at a rate reflecting the 5–7% national prevalence [31], a rate insufficient to achieve the target sample size. Thus, in consultation with biostatisticians, funders, and the Study Monitoring Committee, the study design was modified to enroll the full complement (*n* = 400) of PWOH for primary endpoint analysis of uptake and retention on PrEP at 26 weeks and enroll a smaller number of PWH for observational analysis. The PWH arm would also be randomized to study the conditions but would not be included in primary endpoint analyses (approximately *n* = 40) for descriptive analysis. Dropping the PWH cohort was considered, but it was recognized that effective treatment of HIV contributes critically to prevention by dramatically reducing infectivity, and hence, it will be valuable to know whether the study intervention appears to impact ART uptake among PWH, even in a smaller sample.

Prior work in PWID populations suggests that baseline uptake of MOUD and PrEP in the control condition will likely be modest [19, 32]; correspondingly, power was calculated anticipating a 25% uptake of MOUD and a 5% uptake of PrEP in the control arm. Sample sizes were computed to provide 90% power to detect a 15 percentage point difference in the proportion of participants achieving success, assuming a 5% type I error rate (two-sided). The sample size was computed using a simple two-group test for differences in proportions using quantiles from the standard normal distribution. A sample of 400 participants is needed to detect a 15-point difference in the percentage of MOUD use (25% vs. 40%), and a sample of 216 participants would be needed to confirm a 15-point difference in the percentage of PrEP uptake (5% vs. 20%). To test the intervention effects along PrEP and MOUD outcomes, a total of 400 PWOH are required for testing primary outcome variables. To ensure a diverse participant pool, the study seeks to enroll a minimum of 25% women and a minimum of 25% individuals under 30 years old. Site-specific targets were set for minority racial and ethnic enrollment in the study to reflect the prevalence of persons who inject opioids at each site, as documented by county-level local administrative and epidemiological data.

### Mobile units and study teams

**Fig. 2:**
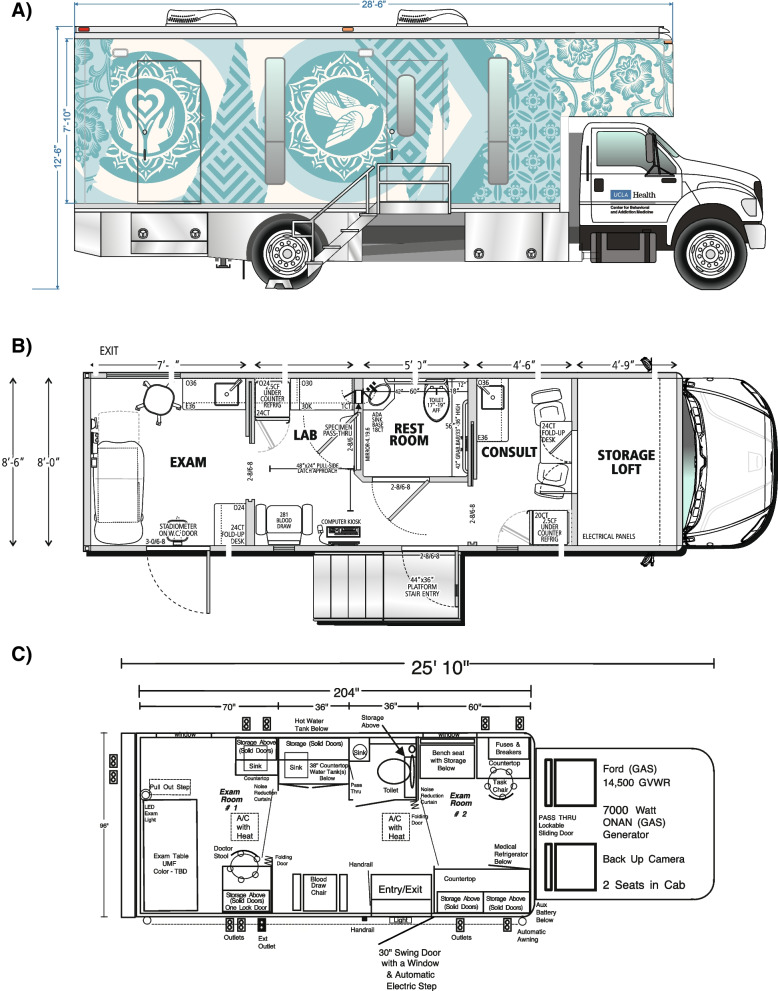
Example of mobile unit and layout. **A** External mock-up of a larger unit. **B** Interior depiction of a larger unit. **C** Interior depiction of a smaller unit

Each site was provided with a mobile vehicle configured as a clinical research space (“mobile unit”). The unit sizes and configurations differ per site preference. Los Angeles, Houston, and New York sites were provided with a 33- by 9-ft mobile unit (LifeLineMobile, Inc., Columbus, OH), whereas Philadelphia and Washington D.C. sites were provided with a 26- by 8-ft mobile unit (Magnum Mobile Specialty Vehicles, Phoenix, AZ). Site preferences differed by city and were guided by the availability of existing mobile units and by concerns over street traffic and parking constraints. All mobile units have an area for medical consultation, an area for blood draw, a toilet, and a separate administrative area. Example mobile unit exterior and interior are depicted in Fig. 2.

Field teams, in general, comprised a clinician (physician, nurse practitioner, or physician assistant), two or three peer navigators and a peer navigator supervisor or field supervisor, a medical assistant, a study coordinator, and a driver. All clinicians completed the necessary training to prescribe buprenorphine (X-waiver, although at the time of publication, an X-waiver is no longer necessary) and have experience in the treatment of addiction and infectious diseases. Navigators were defined as “true peers” if they had been in recovery for OUD for at least a year and had been trained and certified to provide recovery coaching (support and assistance to help others initiate and adhere to MOUD) and health systems navigation. When true peers are not available, the staff members can deliver navigation services in the absence of lived experience, providing they have at least 1 year of education, job experience, or training in delivering navigation services to people living with OUD. All team members were trained during a week-long, national protocol training with additional training in navigation for the peers. Clinicians and peer navigators participate in monthly calls across sites to discuss site-specific issues and potential solutions and for additional training. Field teams are supported (certain roles differ by site) by a site investigator of records, addiction medicine provider or psychiatrist, infectious diseases or HIV provider, project director, qualitative interviewer, pharmacists, couriers, laboratory technicians, regulatory coordinators, outreach coordinator, community engagement experts, and administrators.

### Study procedures

#### Mobile unit locations

Sites utilized a data-driven process to identify potentially viable neighborhoods to place the mobile unit before study initiation leveraging public health surveillance data sources (e.g., overdose data, HIV incidence data) and ecological observations. In pre-implementation, field staff visited locations that are common hot spots, including venue-based areas (locations outside jails, criminal justice community supervision programs, housing shelters, syringe exchange sites, detox centers, and emergency rooms and hospitals), and street-based areas (tourist areas, parks, strolling areas). Viable neighborhoods were expected to change over the course of the study as PWID who are not receiving MOUD are displaced or migrate to new locations due to changes in the local environment (e.g., shifts in policing, gentrification). This data-driven approach was sustained during the course of the study to optimize recruitment and retention activities. In some cities, site locations can be separated by large distances, which requires strategies to support moving the mobile units to prior locations while also planning to place the unit in new locations on a regular basis.

#### Recruitment

Recruitment procedures begin only after consulting with community stakeholders that include police, emergency medical services, local policymakers, business owners, medical and substance use treatment providers, and community advisory boards to inform them of the study and of the proposed placement of the mobile unit. The study team members then canvass areas where the mobile unit will be parked to inform residents and potential participants of the research opportunities in HPTN 094. Flyers and other materials that describe the study are distributed in the surrounding community, as well as to local organizations serving PWID.

#### Screening

**Fig. 3:**
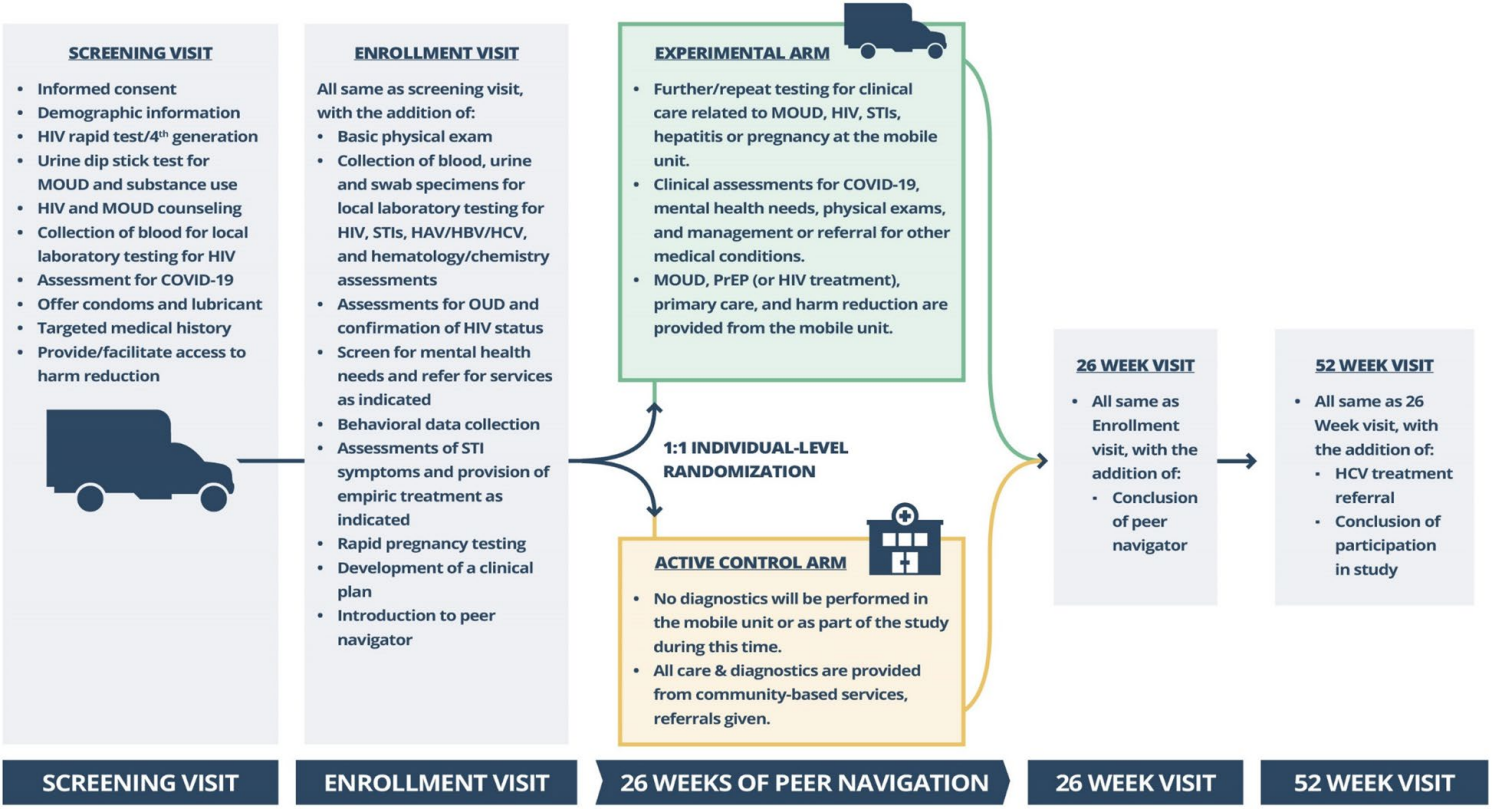
Summary of services provided to the experimental and active control arms

An overview of the study visits and procedures is presented in Fig. 3. Participants are evaluated at baseline and 26-week and 52-week visits. To protect the safety of the community members and staff, sites implemented a pre-screening assessment for COVID-19 before beginning study screening procedures, with those suspected of having COVID-19 being deferred from screening and referred for testing and/or treatment, as appropriate. Sites used an institutional review board (IRB)-approved pre-screening questionnaire to verify if the potential participant meets some of the criteria for the study. If someone is deemed to be potentially eligible, the informed consent process (see Supplement for sample informed consent form) is initiated. The consent form is reviewed by the staff with the study participant, and the participant must complete an assessment of understanding before any screening procedures begin to ensure a full understanding of study activities. Once written informed consent has been obtained, participants begin a screening window (30 days maximum) and, if determined to meet all inclusion and no exclusion criteria (Table 1), enroll in the study at a future date within the screening window. This strategy was devised to enroll participants who have some capacity to return for repeat study visits, which is important to retain participants and to facilitate follow-up visits.

**Table 1:** Study eligibility criteria

***Inclusion criteria***
Adults who meet all of the following criteria are eligible for inclusion in this study:
1. At least 18 years of age^a^
2. Urine test positive for recent opioid use and with evidence of recent injection drug use (“track marks”)
3. Diagnosed with OUD per the Diagnostic and Statistical Manual of Mental Disorders (DSM)-5
4. Able and willing to give informed consent
5. Willing to start MOUD treatment
6. Able to successfully complete an assessment of understanding
7. For people who are not living with HIV: self-reported sharing injection equipment and/or condomless sex in the last 3 months with partners living with HIV or unknown status^b^
8. Able to provide adequate locator information
9. Confirmed HIV status, as defined in the HPTN 094 SSP Manual
***Exclusion criteria***
1. Received MOUD in the 30 days prior to enrollment by self-report
2. Urine testing that is not negative for methadone within 30 days prior to enrollment is exclusionary, unless verified hospital records show methadone received as a medication for hospitalization only during the screening period. A volunteer may provide a sample for urine testing more than once during the screening period in order to achieve a negative result. If this criterion cannot be met within 30 days from the start of screening, the individual will be considered a screen failure and the volunteer has up to two more screening chances to successfully complete the screening process again.^c^
3. Co-enrollment in any other interventional study unless approved by the Clinical Management Committee

Persons who are otherwise eligible to be enrolled will have enrollment deferred if they are suspected to have COVID-19, until they meet the criteria for discontinuation of isolation per CDC guidelines or applicable local guidelines

Prior to December 2021, the following criteria were as follows:

^a^18 to 60 years of age

^b^Applied to both people living with HIV or not living with HIV

^c^Applied as of April 2023

As part of the screening, all participants receive an assessment for eligibility by a clinician in the mobile unit, with assessment for OUD and active injection (observed stigmata), HIV status (rapid point of care tests and laboratory-based 4th generation tests), and screening for opiates and other drugs in urine. All participants are provided with counseling related to MOUD and HIV PrEP and access to harm reduction supplies (naloxone kits and, where possible, syringe services) and STI and HIV prevention kits (e.g., condoms, lubricants) at this and all future visits.

#### Enrollment and randomization

Participants determined to be eligible for enrollment have samples collected for laboratory testing for HIV; hepatitis A, B (surface antibody, core antibody, surface antigen), and C antibody and viral load, if needed (hepatitis B virus DNA or HCV RNA); STIs (chlamydia, gonorrhea, and syphilis); hematology/chemistry (hemoglobin, creatinine, ALT, AST, total bilirubin); pregnancy (if able to become pregnant); and urine drug screening. Those who test positive for HIV receive additional testing (CD4^+^ T cell count, HIV-1 viral load, and resistance testing if appropriate).

At enrollment, behavioral data are collected through an interviewer-led questionnaire and computer-assisted self-interview. Basic medical services are provided to all participants, including empiric treatment for STIs if symptoms are present, provision of harm reduction services (e.g., naloxone); a treatment plan is developed with peer navigators. Participants are assessed for OUD diagnosis per the Diagnostic and Statistical Manual of Mental Disorders, 5th edition (DSM-5) [33] criteria using a clinical interview and checklist. Randomization of participants is performed centrally by the HPTN Statistical Center (SCHARP) via a web-based platform with assignments on a 1:1 basis to the experimental or active control arm. Due to the nature of the intervention, allocation concealment and masking were not possible. Participants and field teams are informed of the study arm immediately after randomization. The study is unmasked at every level (including data analysts). Following the enrollment visit, navigation, counseling, and medical services are provided by the study arm as described below.

#### Study assessments

Primary and secondary endpoints are assessed at the 26- and 52-week visits to test the intervention effects between conditions at week 26 and to evaluate the durability of intervention effects at week 52. At week 26 and week 52 visits, participants complete the behavioral questionnaires and undergo laboratory testing for MOUD and substances of misuse, pregnancy testing if able to become pregnant, PrEP (tenofovir diphosphate via dried blood spots), HIV (for PWOH) and viral load (for PWH), HCV antibodies and viral loads if needed (HCV RNA), and STIs (chlamydia, gonorrhea, and syphilis). A schedule of study assessments is viewable in Table 2.

**Table 2:** Full schedule of assessments

	**Screening**	**Enrollment**	**Care visit(s)**^**1**^	**26 weeks**	**52 weeks**
**Administrative and behavioral procedures**					
Informed consent	X				
Locator information	X	X	X	X	X
MOUD counseling	X	X	X	X	X
HIV risk reduction counseling and test results	X	X	X	X	X
Offer condoms and lubricant	X	X	X	X	X
Provide/facilitate access to harm reduction	X	X	X	X	X
Demographic information	X				
Randomization		X			
Behavioral data collection		X		X	X
Introduction to peer navigator		X			
Conclusion of peer navigation				X	
**Clinical evaluations/procedures**					
Assessment for COVID-19^2^	X	X	X	X	X
Assessment for OUD^3^, recent injection drug use (track marks)^4^	X	X			
Targeted medical history to include MOUD treatment history, HIV risk behaviors, participation in other research studies^5^	X	X	(X)	X^6^	X^6^
Basic physical exam^7^		X	(X)		
Screen for mental health needs and refer for services as indicated		X	(X)	X	X
Initiate (intervention arm) or refer (active control arm) for HIV treatment or PrEP		(X^8^)	(X^8^)		
COWS assessment and initiate mobile unit-based MOUD treatment program (intervention arm only)		(X^9^)	(X^9^)		
Provide clinical management of MOUD and HIV infection or PrEP, including medication or prescription dispensation, as indicated			X		
HAV vaccination referral			X^10^		
HBV vaccination referral			X^10^		
HBV treatment/treatment referral			X^10,11^		
HCV treatment referral			X^10^		X
Development of a clinical plan		X			
Empiric treatment of STIs (if symptomatic)		(X)	(X)	(X)	(X)
Provide lab-based STI results and, if indicated, treatment (intervention arm) or referral (active control arm)			X^12^		
Provide lab-based STI results, and, if indicated, referral				X^12^	X^12^
Provide clinical assessment and management or referral for other medical conditions			X		
Blood collection	X	X	(X)	X	X
Urine collection	X	X	(X)	X	X
Swabs for STI testing^13^		X	(X)	X	X
**Laboratory evaluations/procedures**					
HIV rapid testing	X	X^14^	(X)^15^	X^15^	X^15^
Laboratory-based HIV testing (see SSP manual)	X^16^	X^15^	(X)^15^	X^15^	X^15^
HIV viral load (people living with HIV only)		X	(X)	X	X
CD4 cell count (people living with HIV only)		X	(X)		
MOUD testing (urine)^17^	X	X		X	X
Substance use testing (urine)^18^	X	X	(X)	X	X
Fentanyl testing (urine)	X	X	(X)	X	X
Pregnancy testing (urine)^19^		X	(X)	(X)	(X)
STI testing (syphilis, GC/CT NAAT)		X	(X)	X	X
HCV Ab testing^20^		X	(X)		X
HCV RNA (viral load)^21^		X	(X)	X	X
HBV testing (HBsAg, HbsAb, HbcAb)		X	(X)		
Other HBV-related testing^23^		(X)	(X)		
HAV Ab testing		X	(X)		
Heme/Chem testing^23^		X			
Plasma storage^24^	X	X		X	X
Urine storage^25^		X		X	X
DBS storage (people without HIV only)^26^		X		X	X
Serum storage for SARS-CoV-2 testing^27^		X		X	X

Parentheses around an X indicate that this procedure will be done as needed

See Supplemental material for the explanation of each superscript and additional testing for participants who test positive for HIV

*Abbreviations*: *Ab* Antibody, *ART* Antiretroviral treatment, *aPTT* Activated partial thromboplastin time, *COWS* Clinical Opiate Withdrawal Scale, *DBS* Dried blood spot, *GC/CT* Gonorrhea/chlamydia, *HAV* Hepatitis A virus, *HBV* Hepatitis B virus, *HBcAb* HBV core antibody, *HBsAb* HBV surface antibody, *HBsAg* Hepatitis B surface antigen, *HCV* Hepatitis C virus, *HLA* Human leukocyte antigen, *MOUD* Medications for opioid use disorder, *NAAT* Nucleic acid amplification test, *OUD* Opioid use disorder, *PT* Prothrombin time, *SDMC* Statistical and Data Management Center, *SSP* Study-specific protocol, *STI* Sexually transmitted infection

#### Peer navigation

Participants in both arms work with a trained peer navigator for the first 26 weeks of the study. Peer navigators motivate and assist participants to successfully enroll in, and consistently use MOUD, ART (if PWH), and PrEP (if PWOH), as well as to receive STI testing and treatment, hepatitis vaccine or treatment, SARS-CoV-2 testing (if available/applicable), primary care and harm reduction services. Peer navigators conduct a needs assessment and goal setting at the first navigator encounter and help participants sign up for and access health insurance (Medicaid or other programs for which they are eligible). Depending on participant needs, navigation services include identifying appropriate health service providers in the community, arrangement of medical appointments, reminding participants of upcoming appointments, assisting with transport or accompaniment to appointments, and transferring medical records. Although assistance with obtaining MOUD, adhering to treatment or prophylaxis regimens, and accessing health care services are the primary focus of peer navigation, peer navigators also provide referrals to local resources for participants who have needs related to food security, housing, and employment.

The actual timing, length, and content of navigation visits for each participant are determined by participant needs rather than a fixed study visit schedule. The peer navigation model is guided by a number of overlapping theories: systems theory [34], self-determination theory [35], empowerment theory [36], shared decision-making theory [37], and social support theory [38]. These theories guide how the components of the intervention and the implementation strategies of the navigation are delivered. These theories also emphasize unscripted procedures that include active listening, empathy, and always positive regard with participants. Navigators are trained on the model for 1 week prior to engaging with participants. Fidelity to delivery of the peer navigation model is addressed by conducting monthly supervision sessions to review peer navigation skills. Support to navigators to avoid burnout from constant exposure to trauma from those in active opioid use is provided by navigator supervisors.

#### Experimental arm

Those assigned to the experimental arm receive 26 weeks of MOUD, HIV medication (ART or PrEP), and a limited set of primary care interventions for conditions that can be managed in the mobile unit (a description of services provided is available in Table 3). Although medical and peer navigation services are initially based in the mobile unit, the goal is to successfully transition participants to services at existing facilities in the community by the end of 26 weeks.

**Table 3:** Overview of medical care provided in the mobile unit for intervention arm participants

**Condition**	**Notes**
OUD	MOUD counseling and initiation, management, and dispensing of buprenorphine-based medicine (buprenorphine/naloxone sublingual) Participants who prefer methadone will be referred to community-based services if available
Stimulant use	Participants who also use stimulants (methamphetamine, cocaine) will be referred to 12-step meetings such as crystal meth anonymous, narcotics anonymous and alcoholics anonymous, and evidence-based behavioral treatment, where available.
HIV-ART	ART initiation and management for persons living with HIV who are not already in HIV care, including the following: • Dispensation of one first-line, single-pill regimen to participants for whom this is indicated • Prescription provided for fulfillment at a pharmacy if a different regimen is indicated
HIV-PrEP	PrEP initiation and management for persons without HIV, including the following: • Dispensation of single pill regimens for PrEP • Prescription provided for fulfillment at a pharmacy if a different regimen is indicated
Bacterial STI	Bacterial STI management including the following: • Provision of antibiotics for treatment of bacterial STIs tested for as part of the protocol (chlamydia, gonorrhea and syphilis) • Providing a prescription for fulfillment at a pharmacy, or a referral to community-available services, for any conditions not able to be treated on the mobile unit
Hepatitis C	Testing is part of the study procedures. Those who test positive receive a referral for treatment.
Hepatitis A	Testing is part of the study procedures. Those without evidence of immunity will be referred for vaccination.
Hepatitis B	Testing is part of the study procedures. Those without evidence of immunity will be referred for vaccination. Those with evidence of chronic infection who are living with HIV may be co-treated by an appropriate ART regimen dispensed on the mobile unit or through a prescription fulfilled at a local pharmacy. Those with hepatitis B who are without HIV will be counseled on the risks/benefits of PrEP.
Mental health	Clinicians will screen for symptoms of mental health disorders during visits to the mobile unit. • For any issues identified, participants will be referred for further evaluation and/or care at community-based services, facilitated by peer navigation. • If a person appears to be at risk of harming themselves or others, 911 will be called
Pregnancy	Pregnant participants may utilize the mobile unit. • Participants who are pregnant will continue to be seen for MOUD, ART, PrEP, and other services on the mobile unit. All pregnant participants will be referred for obstetric care with a provider comfortable treating pregnant people who inject drugs treated with MOUD. Study clinicians will endeavor to coordinate with the obstetric care provider to optimize care.
Reproductive health	Limited reproductive health services: • Prescriptions for oral contraception • Condoms/lube
Basic primary care	Basic primary care includes the following: • Diagnosis and management of minor acute illnesses and infections such as upper respiratory tract infections, colds, flu, and diarrheal illness • Providing prescriptions for pharmacy fulfillment for such conditions if indicated. • Identifying more complex care needs and referring for further diagnostics and care from community-available services
More complex care needs and chronic conditions	Management of complex care needs or chronic conditions will be referred to community-available services, such as FQHCs or other clinics Clinicians on the mobile unit may provide prescriptions for medications for chronic conditions that have been lost or stolen or are needed for continuity of care, including communication and coordination, as possible, with the provider managing the care of the chronic condition.
COVID-19	Clinicians will assess participants for COVID-19 at each encounter. Those with suspected COVID-19 or recent exposure will be referred for further evaluation, care, and treatment, as appropriate and available. CDC and local guidelines for discontinuation of isolation will direct when participants with suspected COVID-19 or recent exposure can resume in-person visits. Distance procedures to collect data and monitor health will be implemented to the extent possible.

*Abbreviations*: *ART* Antiretroviral therapy, *FQHC* Federally qualified health centers, *MOUD* Medications for opioid use disorder, *OUD* Opioid use disorder, *PrEP* Pre-exposure prophylaxis, *STI* Sexually transmitted infection

Participants assigned to the experimental arm are provided immediate access to MOUD to minimize the disorganizing behavioral effects of opioid addiction that jeopardize initiation and adherence to medication for HIV prevention and care. The MOUD regimen provided in the mobile unit is influenced by the participant’s recent drug use and treatment history, the drug regimens used in local opioid treatment programs, and the availability of medications on the mobile unit. Sublingual buprenorphine-based medication is available on the mobile unit or via prescription based on local site implementation; methadone is available via navigation/linkage to external opioid treatment programs.

For PWOH, HIV risk reduction counseling is provided and participants are offered oral PrEP. Descovy (emtricitabine/tenofovir alafenamide fumarate, Gilead Sciences) and Truvada (emtricitabine/tenofovir disoproxil fumarate, Gilead Sciences) are available on the mobile unit. Descovy is offered to those assigned male at birth, while Truvada is offered to those assigned female at birth. PrEP regimens can also be prescribed, including long-acting injectables, though not provided by this study. Participants are tested for HIV during the intervention period per PrEP guidelines (and additional times if requested). For PWH and those who acquire HIV during the study, ART is provided in the mobile unit or encouraged to continue with their current HIV provider if already engaged in care. Biktarvy (bictegravir/emtricitabine/tenofovir alafenamide, Gilead Sciences) is available on the mobile unit, but other ART regimens can be prescribed.

Participants receive additional services either on the mobile unit or by meeting with their navigators to receive facilitated referrals to appropriate services delivered in the community. Participants with STI symptoms are offered treatment empirically on the mobile unit. Treatment or referrals (for cases that cannot be addressed in the mobile unit) are provided for those with laboratory-confirmed STI diagnoses that are otherwise asymptomatic. Assessment and/or treatment of some other medical conditions is provided in the mobile unit, whereas treatment or management of complex conditions requires a referral. Participants identified to have active chronic HCV are referred for treatment with external providers. Experimental arm participants may receive some or all of their care from community-based services instead of the mobile unit (for example, if they are already receiving HIV-care from a clinic in the community) if preferred by the participant.

#### Active control arm

As there is only a limited set of standard services available for PWID and at risk for HIV in the US, ethical considerations required the development of an active control condition. Participants assigned to the active control condition receive 26 weeks of peer navigation identical to peer navigation provided to the experimental arm, with the exception that peer navigation sessions occur in spaces away from the medical unit or virtually for the active control arm. Navigation services provided to active control participants are designed to link participants to the comparable medication and services for MOUD, PrEP, STIs, HCV, and primary care as provided for the experimental participants in the mobile unit. The key difference is that navigators link participants to services provided at existing, local community agencies.

#### Retention

Each study site targets a retention rate of at least 90% at week 26 and 52 visits. Sites developed local retention plans seeking input from Community Advisory Boards (CABs). In general, sites collect detailed locator information at study screening and actively review and update this information at subsequent contacts, provide a thorough explanation of the procedural requirements and the importance of the two arms to the overall success of the study at each visit, use appropriate and timely visit reminder strategies (e.g., personalized calendars or post cards, electronic reminders, social media platforms, and phone calls), mobilize trained outreach staff to complete in-person contact with participants at their homes or other community locations, may provide contact incentives to promote engagement in-between visits, and regularly communicate with the community at large (e.g., service providers, CABs).

An intent-to-treat analysis is intended, and participants remain in their randomized arm for the duration of the study. Participants may voluntarily withdraw from the study for any reason at any time. The investigator of record at each site or designee also may withdraw participants from the study in order to protect their safety and/or if they are unwilling or unable to comply with required study procedures. Additionally, participants also may be withdrawn if the study sponsor, government or regulatory authorities, or IRB terminate the study prior to its planned end date.

At the conclusion of the 26-week visit, all care activities are directed to community partners. The transition from the intervention (particularly for the mobile unit arm) to community-based services will occur prior to the 26-week visit as much as possible to ensure a smooth transition.

### Data management

Study case report forms (CRFs) and other study instruments are developed by the protocol team and HPTN Statistical Data Management Center (SDMC). Data are collected using a validated web-based software and are submitted to the HPTN SDMC for cleaning, reporting, and analysis. Quality control data queries are generated on a routine schedule for verification and resolution by the site data management staff. Rates of accrual, adherence, follow-up, and serious adverse event (SAE) incidents are monitored closely by the team as well as the HPTN Study Monitoring Committee (SMC). A subset of the protocol team, the Clinical Management Committee (CMC), addresses issues related to study eligibility and SAE management and reporting as needed to assure consistent case management, documentation, and information-sharing across sites.

The SDMC utilizes system and programmed edit checks within the electronic database as well as the Integrated Data Review Plan (IDRP) to specify the data to review and clean to ensure the quality of the data recorded/captured for study subjects on an ongoing basis. In the context of this overall accountability, other study team members bear function-specific responsibility to ensure data quality based on their areas of expertise and additional role-specific tasks. For example, the SDMC Clinical Safety Associate monitors SAE data for completeness and consistency. Specifics regarding monitoring, parameters, timing, and reports are documented in the study-specific data management plan, safety management plan, and statistical management plan.

In addition to SDMC data quality procedures, study site monitoring is performed by the DAIDs monitoring branch using a target source data verification plan. Specifications are developed in consultation with the sponsor. After all data have been reviewed by the SDMC and DAIDS monitoring branch, each site investigator or designee must sign off on each participant’s complete set of data to attest that the data has been reviewed and is deemed to be accurate. When all data collection and verification procedures are complete, the SDMC will lock the database, and no further changes can be made.

### Data analysis

Consistent with the primary study objective to evaluate whether the intervention improves the use of MOUD and increased use of PrEP among PWOH, as measured at 26-week visits, the following endpoints will be assessed:

- A positive MOUD outcome is defined as being alive, retained, having biological evidence of MOUD (any detectable medications) at the week 26 visit, and having a verified MOUD prescription or verified enrollment in an opioid treatment program and current at the 26-week visit.
- A positive HIV prevention outcome is defined as being alive, retained, HIV-negative, and having detectable PrEP drugs in dried blood spot samples at the week 26 visit.

The primary outcome will be analyzed under the intention-to-treat principles. Participants not present to provide data at 26-week visits will be assumed to not have met the criteria for MOUD and PrEP endpoints, which enables all enrolled participants to be included in the primary analysis, regardless of loss to follow-up or death. All endpoint proportions will be compared across study arms using simple linear-binomial regression models (estimating risk differences) adjusted for site (strata variable). No interim analyses are planned.

Multiple secondary outcomes are pre-specified and described in Supplemental Table 1. Secondary analysis endpoint proportions will be compared using the identical method described for the comparison of proportions among primary endpoints with the exception of within-cohort change over time. Within-cohort change over time will be estimated using correlated-data binomial regression (a random-effects model estimating risk differences) adjusted for city. Outcome incidence rates will be computed as the number of incident events (including recurrent events where applicable) divided by total number of accrued person-years in each study arm, where person time is computed for each person as the difference between enrollment date and the last available sample-collection date for which the corresponding STI test was performed. Person-time for participants with new HIV infection will be calculated as the difference between the enrollment date and the date of the first detection of HIV infection.

Because of the unmasked nature of the trial at every level, all investigators and staff members for the trial were not allowed access to any variables measuring primary outcomes and most secondary outcomes. All biological measures of outcomes were unavailable throughout the trial progress. Also, tallies and analysis of reported outcomes that involved primary and secondary outcomes were unavailable to investigators and staff members for the trial. Data and safety monitoring procedures for the trial involved evaluation of reported outcomes, especially overdose, fatalities, hospitalizations, and HIV seroconversions by condition. These reports were available only to members of the SMC for this study and were withheld from study investigators and staff members.

### Implementation science components of the study

A concurrent, embedded, mixed-methods approach is used to collect quantitative and qualitative data from each study site to document facilitators and barriers associated with implementation fidelity, and effectiveness and to inform future dissemination of integrated healthcare delivered using medical units. Data collection is theory-driven, guided by the Practical, Robust, Implementation, and Sustainability Model [39], and includes three primary sources: documentation of protocol activities, ethnographic observations, and semi-structured in-depth interviews all initiated at the time of implementation of the randomized trial.

#### Pre-implementation phase

During pre-implementation of the study protocol at the different sites, study personnel conducted preparatory activities to engage with community members and leaders and build partnerships with public health agencies, and community providers of medical, mental health, substance use, and ancillary services. Sites developed and engaged regularly with their local CABs to provide input from community members with lived experience and those committed to improving the health of PWID. CABs advise on issues regarding cultural competence for the study teams and provide suggestions for sustained recruitment and retention strategies. During start-up, sites documented the baseline status of the local opioid and HIV crises, and the policies and agencies aligned to address these, primarily by using local administrative and epidemiological datasets. Observational fieldwork in areas provided landscape data advising optimal areas to park the mobile medical units and to identify existing, site-specific agencies and clinics that provide services for addiction, HIV, and primary care for PWID.

**Fig. 4:**
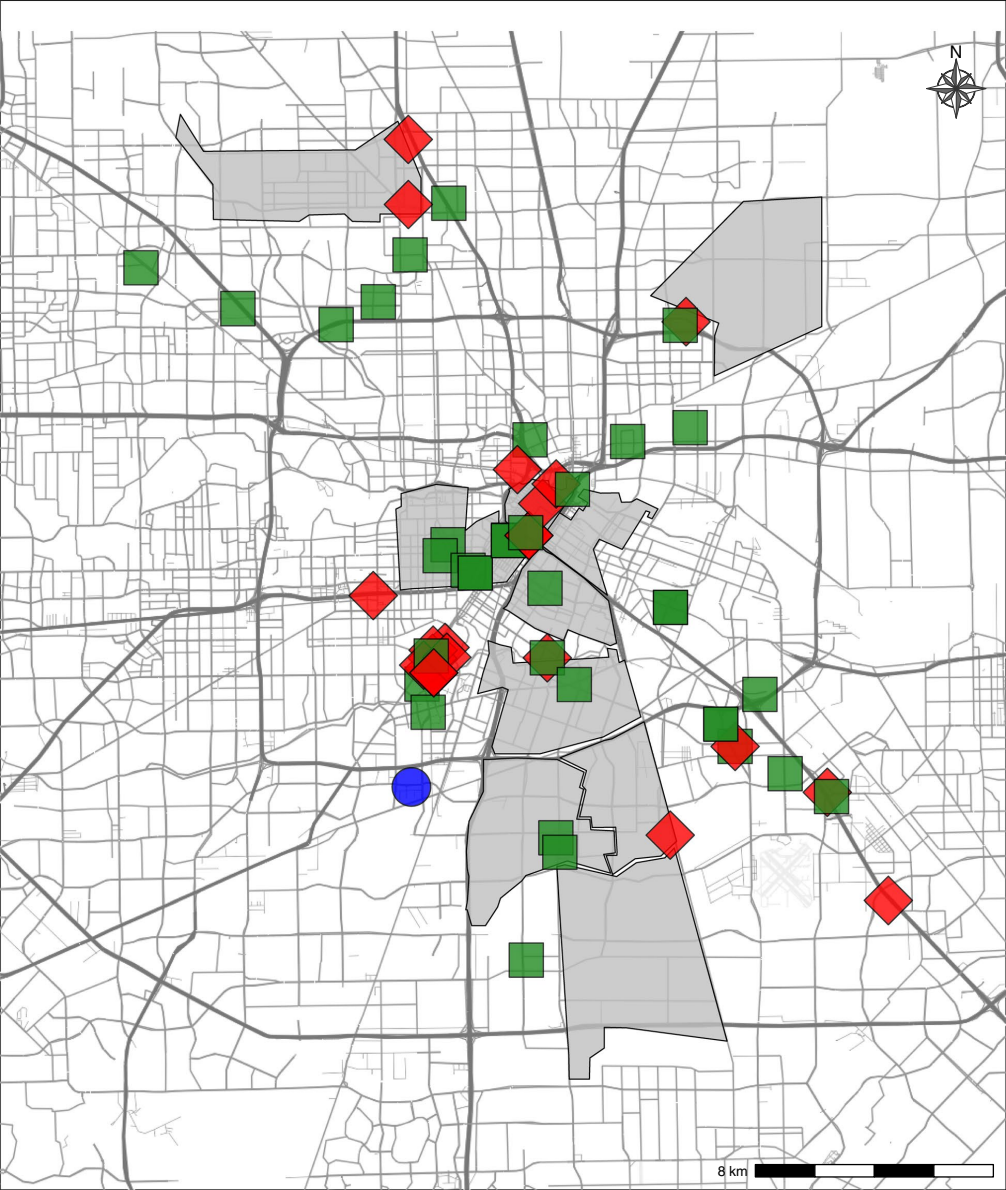
Example landscape analysis. The blue marker identifies where the mobile unit is parked when not in use; red markers are emergency rooms, jails, or prisons; green markers are community-based services for primary care clinics, HIV care, MOUD, syringe service programs in the Houston metropolitan area. The shaded areas identify areas with significant opioid overdose

A one-time formal landscape analysis was used to document the objective criteria for selecting potential neighborhoods in which intervention services and participant recruitment should occur. Contextual data of each neighborhood were captured which include the areas’ demographics, HIV incidence, overdose rate, and drug-related arrests. Additionally, sites collected geolocated data related to overnight parking location of the mobile unit, daily activity sites, emergency rooms, jails and prisons, and other brick-and-mortar services (e.g., primary care clinics, HIV care, MOUD, syringe service programs, overdose prevention distribution). These data were then used to create a map of neighborhoods where the mobile unit would provide services (example in Fig. 4).

#### Implementation phase

During the implementation phase of the study, a data-driven approach was used to identify additional viable neighborhoods for potential recruitment. Outreach activities are conducted in such neighborhoods to raise the profile of the study and the study staff members in order to build rapport and trust with individuals in the community of PWID. Community members and participants are kept informed of the locations (e.g., palm cards, websites, text messaging, word of mouth) where the mobile unit will be parked and of the operating times. Individuals engage with the staff members on the mobile unit to receive harm reduction services (naloxone distribution and syringe services, if permissible) and educational information regardless of participation in the study.

Protocol activities are captured by the implementation science evaluation team by documenting the process and outcomes of study-related meetings and documenting the conduct of the study and contexts informing adaptations to the implementation of integrated healthcare delivery [40]. Participant data are examined relative to the racial and ethnic composition of recruitment neighborhoods to assess the reach the intervention team has into the local communities of individuals with minority racial and ethnicity identities.

An ecological assessment of implementation neighborhoods is completed weekly by the site team members to determine contemporaneous changes in locations where PWID frequent and any notable characteristics of—or changes to—the neighborhood site where the mobile unit was located that may have affected participant enrollment or treatment. Additionally, sites report any notable changes to the study mobile unit or study resources (e.g., equipment, staff) that may have affected how treatment was delivered to participants or accessed by participants.

Finally, semi-structured, in-depth interviews identify and contextualize barriers and facilitators to the implementation of the intervention that may influence primary and secondary outcomes. Interviews are being conducted with PWID (*n* = 17 per site), intervention providers and staff (*n* = 7 per site), and community stakeholders (*n* = 15 per site) to elicit multi-level factors (i.e., patient, healthcare delivery, community environment, structural) affecting the intervention delivery, care access, and ability to sustain engagement in integrated care services and to community-based services following week 26.

### Cost and impact analysis

In the cost-effectiveness analysis, the per-participant costs of implementing the HPTN 094 package of interventions will be assessed taking a payer perspective and utilize published cost data, internal reports, and supply-chain data to determine the intervention package’s incremental cost compared to the control condition. We will also report the total non-research costs of the intervention package. Medical resource utilization by participants of trial-provided services will be tracked internally. Estimates of differential medical resource utilization of outside-of-trial services by trial arm will be estimated through participant self-reports. Medical resource costs both incurred and averted by assignment to the trial arm will be incorporated. Based on the intervention effect measured by the primary trial endpoint, the incremental cost-effectiveness ratio of providing the intervention package over the control condition will be estimated in terms of dollars per-person meeting the combined composite endpoint of being alive, retained, using an MOUD, HIV-negative, and having detectable PrEP in their dried blood spot at week 26. This analysis uses combined costs as the cost-effectiveness of “on MOUD” and a separate cost-effectiveness of “on PrEP” cannot account for overlapping costs that are incurred and are inseparable.

In the impact modeling analysis, we will construct a stochastic individual-based cohort model that will provide an estimate of intervention impact on the individual-level risk of HIV acquisition among PWOH for cohorts of PWID representative of the trial populations in the study sites. The model will be parameterized and calibrated for each study site using localized epidemic and care cascade data. Demographic, behavioral, and injection drug use data collected at baseline and during the trial will be used to model the sexual activity and needle-sharing behavior of the trial participants and their exposure to HIV. Adherence to interventions (including MOUD and/or PrEP), injection drug use, and sexual risk behaviors will be simulated. The model will be parameterized with both local data and the individualized data collected during trial visits.

Utilizing the impact modeling results, the cost and effectiveness estimates with respect to MOUD and PrEP retention will be extrapolated to incremental cost per HIV acquisition averted over a 10-year horizon. If supported by evidence of effectiveness from secondary endpoints, incremental costs per fatal and non-fatal overdose will also be reported.

### Data safety and monitoring

A SMC conducts interim reviews of study progress, including rates of participant accrual, visit retention, and completion of primary and secondary endpoint data collection. The SMC is kept apprised by the study team of contemporary changes in PWID and how such changes may affect study-monitoring metrics. Examples include the dominance of fentanyl availability in the street opioid market, polysubstance use, and the emergence of xylazine in the street drug supply at sites. In a closed report, the SMC reviews safety data by arm.

Adverse events are recorded in source documentation, and only serious adverse events are entered into study databases and assessed for relatedness to study products by site study clinicians. Grading of the severity of events is reported based on guidelines in the DAIDS Table for Grading the Severity of Adult and Pediatric Adverse Events, corrected version 2.1, July 2017 [41]. Suspected unexpected serious adverse reactions (SUSAR) are reported in an expedited manner. Additionally, social impacts of participating in the study (e.g., participants could be perceived as having HIV or at high risk for acquiring HIV or could be subject to stigma related to their HIV status or their use of injection drugs) are collected and submitted to the study database during regular visits.

The SMC meets at least annually to review safety and efficacy data. More frequent or ad hoc reviews of safety data may be conducted by the SMC as needed. The SMC may make recommendations based on a review of safety and efficacy data.

An external monitoring agency (PPD) visits the sites quarterly to verify compliance with human subjects and other research regulations and guidelines; assess adherence to the study protocol, study-specific procedures manual, and local counseling practices; and confirm the quality and accuracy of information collected at the study site and entered into the study database. Site investigators allow study monitors to inspect study facilities and documentation, observe the performance of study procedures, and inspect all study-related documentation by authorized representatives.

### Ethics

The protocol, site-specific informed consent forms, participant education and recruitment materials, and any subsequent modifications are reviewed and approved by the HPTN Scientific Review Committee, the NIAID Prevention Science Review Committee, and a central IRB responsible for oversight of this research study. The IRB reviews the protocol at least annually, and investigators submit safety and progress reports to the IRB at least annually. All study-specific activities commenced after approval by these regulatory bodies.

Written informed consent was obtained from each study participant by designated personnel at each site (e.g., investigator of records or study clinicians). Additionally, sites may have additional consent requirements based upon local requirements (e.g., for clinical care, privacy protection, state-specific bill of rights). Participants have additional options to consent to the storage of blood or urine for future research or be contacted for future studies.

All study-related information is stored securely in locked cabinets or password-protected electronic systems. All laboratory specimens, reports, study data collection, process, and administrative forms are identified by a coded number only to maintain participant confidentiality. All databases are secured with password-protected access systems. Forms, lists, logbooks, appointment books, and any other listings that link participant ID numbers to other identifying information are stored in a separate, locked file in an area with limited access.

Interviews are transcribed by qualified personnel, and all identifiable information is removed from the transcripts. The HPTN obtained a Certificate of Confidentiality from the US Department of Health and Human Services applicable to this study. This certificate protects the study staff from being compelled to disclose study-related information by any federal, state, or local civil, criminal, administrative, legislative, or other body.

Of note, there is no form or mechanism of compensation for those who suffer harm from trial participation.

### Dissemination policy

Reporting of trial results (primary endpoint data) is planned within a conference setting with nearly simultaneous, coordinated press releases from the HPTN, the sponsor (DAIDS), and the funder (NIDA). HPTN and the study team will work to present the information to the study community groups before the official release of the results. Before this release of the primary results, no abstract or manuscript may be published which includes primary or secondary endpoint results. The study employs a Protocol Publications Committee (consisting of protocol team members) which triages manuscript concepts to tier the submissions. Higher-tiered concepts move forward before lower-tiered concepts to best utilize the available staffing resources. Anyone may propose a manuscript but for author eligibility for a manuscript the HIV Prevention Trials Network (HPTN) adheres to the “Recommendations for the Conduct, Reporting, Editing, and Publication of Scholarly Work in Medical Journals” Section II.A “Authorship and Contributorship” set forth by the International Committee of Medical Journal Editors [42].

Once reporting on the primary and secondary endpoints is complete, the SDMC will finalize the creation of de-identified public-use datasets, data dictionaries, and other supporting documentation, which will be uploaded to the relevant data repositories (about 2 years after the last study visit). This information will be located on an HPTN Public Data Access page and access will be granted after the completion of a short application.

### Trial status

The HPTN 094 study commenced recruitment at the different sites between May and June 2021. Enrollment was completed as of September 2023. The final study visit is expected before September 2024. Version 2.0 of the protocol was approved by the IRB on March 27, 2023, and implemented by the study sites thereafter.

## Discussion

Development and initial implementation of this protocol to evaluate the impact of integrated strategies to deliver MOUD, HIV prevention, STI treatment and testing, HCV testing, and primary care in a mobile health delivery unit compared to peer navigation to existing services in the community in five US cities already is yielding important observations. The mobile unit provides outreach in the community and serves as a short-term (up to 26 weeks) bridge to community-based services. The work to this point in designing and implementing this protocol underscores the multiple and intersecting health problems for people who inject opioids who are not in medication treatment that include the following: (1) being at risk for HIV acquisition or untreated HIV disease; (2) living with undetected and untreated STIs; (3) having poor care for physical health conditions; (4) facing severe barriers to accessing many aspects of social determinants of health, particularly poverty, and housing and food insecurity; (5) having a multitude of infectious complications related to unsterile injection; (6) high risk of death by overdose; and (7) high risk of suffering physical trauma or violence. As such, the protocol brings into sharp relief a forgotten group in the US who face multiple challenges to health on a daily basis. In the national setting of leveraging resources and energies to develop and scale up vaccines for COVID-19, we have found the health quality of PWID out of treatment for OUD and living with or at risk for HIV to be disproportionately poor. Evidence for this statement includes the need to develop and to implement the peer navigation condition for all participants in this study as an ethical requirement to conduct the study as there are no existing services that might serve as “treatment as usual” for this group anywhere in the country.

The study will answer the question of whether the evidence supports using a mobile health unit to deliver the range and intensity of integrated services to PWID to improve health, including HIV prevention outcomes, though for PWH, the small sample of participants will support only descriptive analyses. Low-to-moderate quality studies show that mobile units improve care outcomes in multiple settings and for multiple health needs. Needle exchange programs delivered from mobile units have been successful in engaging PWID in services that are either unacceptable or unavailable in brick-and-mortar settings [43, 44]. These prior studies provide a key rationale for evaluating this experimental approach to the delivery of integrated services in this study. Independent of the outcome of whether the mobile unit proves superior in outcomes over peer navigation to existing services in communities, the observations of the team in developing the protocol and implementing the project in the five US cities emphasize the need for an increase in attention by stakeholders and public health policymakers to address the critical health emergency within this neglected group of Americans.

A related finding from the implementation of the protocol is that among PWH, we were unable to find and enroll sufficient numbers of individuals who are currently injecting opioids and not in treatment to fully power testing of the proposed prospective hypotheses for this group, despite multiple and targeted efforts to do so. One reason for this could be that PWID with HIV died earlier in the HIV epidemic and are unavailable. Life expectancy for PWH has increased dramatically since the introduction of ART in MSM and other risk groups nearing that of PWOH. By contrast, life expectancy for PWID has remained stagnant at close to 30 years for someone diagnosed with HIV at age 20 [45, 46]. It is also possible that providing health care and supportive services for PWH via the Ryan White Care Act may have improved access to “whole-person” treatments, including those for addiction, where indicated. In support of this idea, in 2018, 74% of PWH reported an HIV-related visit to a healthcare provider within the prior 6 months, 70% reported currently taking medicines to treat HIV, and 50% reported receiving MOUD [13]. Lessons learned from the provision of integrated healthcare and supportive services for PWH in the HIV era may show the way forward to address the intertwining conditions experienced by people with OUD without HIV [11].

The implementation science activities that are integral to the HPTN 094 study will provide essential procedural data for using medical units in urban areas to address OUD and HIV outcomes for PWID. These lessons will be different from those needed for PWID in rural areas but may include describing the needs for creative approaches that can be used to solve space issues (e.g., parking the mobile unit next to a facility that can provide quiet and private spaces for navigation sessions). Also, findings will address logistical challenges (e.g., portable generators to maintain air conditioning, refrigeration, and other requirements, using locations that provide public access to WiFi for consistent Internet access, etc.) that are intensified by a range of community and environmental challenges (violence, COVID-19, weather extremes).

One early observation from the implementation science activities has been that participants seen on mobile units frequently are themselves mobile. For participants experiencing homelessness, moving the unit even a few blocks or to locations other than where contact was initially made and treatment first received can affect whether and if follow-up visits are completed. The implementation protocol utilizes a contextual data-driven approach to systematically inform the decisions about where to place the mobile units, how frequently to return to specific locations, and when to move on to new locations when the availability of new potential participants is exhausted.

To frame the study outcomes of quantitative analysis of primary and secondary outcomes, this study has built-in comprehensive costing and cost-effectiveness analyses. The peer navigation provided in the active control arm is intended to equal what is provided in the experimental arm; this will allow us to evaluate the marginal effects of integrated care in the mobile unit supported by navigation compared to navigation to community-based services without direct provision of care. We may find that most of the benefit seen in the experimental arm is due to peer support and navigation alone; this would provide policymakers with useful cost-effectiveness data. Important data will also come from the analysis of outcomes at 52-week visits, measuring the durability of treatments through 6 months after the conclusion of the interventions.

Lastly, if the mobile unit intervention proves to be superior, documentation of this effect in a randomized controlled trial will be helpful for integrating this intervention into public health guidelines in the presence of active peer navigation. This study will provide valuable information to policymakers in the four main areas that have been recognized as presenting substantial limitations to using mobile health clinics to deliver integrated care services: (1) fragmentation of care, (2) financial issues, (3) spatial and structural limits, and (4) logistical challenges. The findings from this research will directly address the fragmentation of care by providing integrated health services. Moreover, findings from the implementation science components of this study will provide data on strategies of recruitment of participants, retention, and also on barriers and facilitators that guide and for the implementation of the integrated mobile unit.

The importance of this study is highlighted by the fact while some investigators pioneered work on MOUD as HIV prevention for people who inject opioids early in the epidemic [47], HPTN 094 is the first study implemented in the US by the large, HIV science networks to evaluate interventions to improve HIV prevention outcomes for PWID. Evaluation of MOUD in international settings shows improvement in HIV outcomes for people who inject opioids [19]. The contrast between effects observed for MOUD in PWH internationally cannot be confirmed in this study, as we are unable to enroll sufficient numbers of participants living with HIV in the US to test the question prospectively. By contrast, as the overdose crisis continues in the US and HIV incidence is again rising in this group at behavioral risk for a wide range of negative health consequences, including HIV incidence, mortality, and significant morbidity, we have learned as a team that independent of study outcomes for HPTN 094, it is likely we will show medications are insufficient as a means to treat our way out of the opioid and the HIV crises. Testing integrated healthcare and supportive services to PWID via a mobile unit may be a key way to improve health and to prevent disease transmission among this vulnerable population. This protocol will provide important results in addressing intertwining epidemics.

### Supplementary Information

**Additional file 1.** Sample informed consent form.

**Additional file 2: Supplemental Table 1.** WHO Trial Registration Data Set (Version 1.3.1). **Supplemental Table 2.** Footnotes for Table 2. **Supplemental Table 3.** Additional procedures for participants who have a reactive or positive HIV test after enrollment.

**Additional file 3.** SPIRIT 2013 Checklist: Recommended items to address in a clinical trial protocol and related documents.

## Data Availability

Data collection for this study is ongoing at the time of publication; as such, no data are available. Protocol and letter of amendment files are available at https://www.hptn.org/research/studies/hptn094.

## Abbreviations

ART: Antiretroviral therapy
CAB: Community Advisory Boards
DSM-5: Diagnostic and Statistical Manual of Mental Disorders, 5^th^ edition
HCV: Hepatitis C virus
HPTN: HIV Prevention Trials Network
IRB: Institutional Review Board
MOUD: Medications for opioid use disorder
MSM: Men who have sex with men
NIAID: National Institute of Allergy and Infectious Diseases
NIH: National Institutes of Health
NIDA: National Institute on Drug Abuse
OUD: Opioid use disorder
PWID: People who inject drugs
PWH: People with HIV
PWOH: People without HIV
PrEP: Pre-exposure prophylaxis
STI: Sexually transmitted infection
SMC: Study Monitoring Committee
SCHARP: HPTN Statistical Center
US: United States

## References

[1] Ahmad FB, Cisewski JA, Anderson RN (2022). Provisional mortality data—United States, 2021. MMWR Morb Mortal Wkly Rep.

[2] Kariisa MDN, Kumar S (2022). Vital signs: drug overdose deaths, by selected sociodemographic and social determinants of health characteristics—25 states and the District of Columbia, 2019–2020. MMWR Morb Mortal Wkly Rep.

[3] Ciccarone D (2021). The rise of illicit fentanyls, stimulants and the fourth wave of the opioid overdose crisis. Curr Opin Psychiatry.

[4] Spetz J, Hailer L, Gay C, Tierney M, Schmidt L, Phoenix B (2022). Changes in US clinician waivers to prescribe buprenorphine management for opioid use disorder during the COVID-19 pandemic and after relaxation of training requirements. JAMA Netw Open.

[5] Amram O, Amiri S, Panwala V, Lutz R, Joudrey PJ, Socias E (2021). The impact of relaxation of methadone take-home protocols on treatment outcomes in the COVID-19 era. Am J Drug Alcohol Abuse.

[6] Volkow ND, Frieden TR, Hyde PS, Cha SS (2014). Medication-assisted therapies—tackling the opioid-overdose epidemic. N Engl J Med.

[7] Volkow ND, Jones EB, Einstein EB, Wargo EM (2019). Prevention and treatment of opioid misuse and addiction: a review prevention and treatment of opioid misuse and addiction prevention and treatment of opioid misuse and addiction. JAMA Psychiatry.

[8] Mauro PM, Gutkind S, Annunziato EM, Samples H (2022). Use of medication for opioid use disorder among US adolescents and adults with need for opioid treatment, 2019. JAMA Netw Open.

[9] Krawczyk N, Rivera BD, Jent V, Keyes KM, Jones CM, Cerdá M (2022). Has the treatment gap for opioid use disorder narrowed in the U.S.?: a yearly assessment from 2010 to 2019. Int J Drug Policy.

[10] Dong H, Stringfellow EJ, Russell WA, Jalali MS. Racial and ethnic disparities in buprenorphine treatment duration in the US. JAMA Psychiatry. 2023;80(1):93–5. 10.1001/jamapsychiatry.2022.3673. 10.1001/jamapsychiatry.2022.3673 PMC9647560 36350592

[11] National Academies of Sciences E, and Medicine (2020). Opportunities to improve opioid use disorder and infectious disease services: integrating responses to a dual epidemic.

[12] Harvey L, Boudreau J, Sliwinski SK, Strymish J, Gifford AL, Hyde J (2022). Six moments of infection prevention in injection drug use: an educational toolkit for clinicians. Open Forum Infect Dis.

[13] Handanagic S, Finlayson T, Burnett JC, Broz D, Wejnert C (2021). HIV infection and HIV-associated behaviors among persons who inject drugs - 23 metropolitan statistical areas, United States, 2018. MMWR Morb Mortal Wkly Rep.

[14] Rosenberg ES, Rosenthal EM, Hall EW, Barker L, Hofmeister MG, Sullivan PS (2018). Prevalence of hepatitis C virus infection in US states and the district of Columbia, 2013 to 2016. JAMA Netw Open.

[15] Platt L, Easterbrook P, Gower E, McDonald B, Sabin K, McGowan C, Yanny I, Razavi H, Vickerman P (2016). Prevalence and burden of HCV co-infection in people living with HIV: a global systematic review and meta-analysis. Lancet Infect Dis.

[16] Williams LD, Ibragimov U, Tempalski B, Stall R, Satcher Johnson A, Wang G (2020). Trends over time in HIV prevalence among people who inject drugs in 89 large US metropolitan statistical areas, 1992–2013. Ann Epidemiol.

[17] Des Jarlais DC, Sypsa V, Feelemyer J, Abagiu AO, Arendt V, Broz D (2020). HIV outbreaks among people who inject drugs in Europe, North America, and Israel. Lancet HIV.

[18] Strathdee SA, Kuo I, El-Bassel N, Hodder S, Smith LR, Springer SA (2020). Preventing HIV outbreaks among people who inject drugs in the United States: plus ça change, plus ça même chose. AIDS.

[19] Miller WC, Hoffman IF, Hanscom BS, Ha TV, Dumchev K, Djoerban Z (2018). A scalable, integrated intervention to engage people who inject drugs in HIV care and medication-assisted treatment (HPTN 074): a randomised, controlled phase 3 feasibility and efficacy study. Lancet.

[20] Bartholomew TS, Andraka-Cristou B, Totaram RK, Harris S, Doblecki-Lewis S, Ostrer L (2022). “We want everything in a one-stop shop”: acceptability and feasibility of PrEP and buprenorphine implementation with mobile syringe services for Black people who inject drugs. Harm Reduct J.

[21] Peters PJ, Pontones P, Hoover KW, Patel MR, Galang RR, Shields J (2016). HIV infection linked to injection use of oxymorphone in Indiana, 2014–2015. N Engl J Med.

[22] Golden MR, Lechtenberg R, Glick SN, Dombrowski J, Duchin J, Reuer JR (2019). Outbreak of human immunodeficiency virus infection among heterosexual persons who are living homeless and inject drugs - Seattle, Washington, 2018. MMWR Morb Mortal Wkly Rep.

[23] Cranston K, Alpren C, John B, Dawson E, Roosevelt K, Burrage A (2019). Notes from the field: HIV diagnoses among persons who inject drugs - northeastern Massachusetts, 2015–2018. MMWR Morb Mortal Wkly Rep.

[24] Centers for Disease Control and Prevention (CDC). HIV surveillance report, 2014. 2015.

[25] (CDC) CfDCaP. HIV surveillance report, 2019. 2021. p. 32.

[26] Centers for Disease Control and Prevention. Core indicators for monitoring the Ending the HIV Epidemic initiative (preliminary data): National HIV Surveillance System data reported through September 2022; and preexposure prophylaxis (PrEP) data reported through June 2022. 2022. Available from: https://www.cdc.gov/hiv/library/reports/surveillance-data-tables/.

[27] Streed CG, Morgan JR, Gai MJ, Larochelle MR, Paasche-Orlow MK, Taylor JL (2022). Prevalence of HIV preexposure prophylaxis prescribing among persons with commercial insurance and likely injection drug use. JAMA Netw Open.

[28] El-Bassel N, Marotta PL, Gilbert L, Wu E, Springer S, Goddard-Eckrich D, et al. Integrating treatment for opioid use disorders and HIV services into primary care: solutions for the 21st century. In: Crosby RA, DiClemente RJ, (eds), Structural interventions for HIV prevention: optimizing strategies for reducing new infections and improving care. New York: Oxford Academic; 2018. 10.1093/oso/9780190675486.003.0008.

[29] Williams AR, Nunes EV, Bisaga A, Pincus HA, Johnson KA, Campbell AN (2018). Developing an opioid use disorder treatment cascade: a review of quality measures. J Subst Abuse Treat.

[30] Primm B (1991). Overview of the Office for Treatment Improvement and its philosophy. J Psychoactive Drugs.

[31] Centers for Disease Control and Prevention (CDC). HIV surveillance report, 2020. Atlanta; 2020 [11-15-2022]. Available from: https://www.cdc.gov/hiv/library/reports/hiv-surveillance.html.

[32] Morgan JR, Schackman BR, Leff JA, Linas BP, Walley AY (2018). Injectable naltrexone, oral naltrexone, and buprenorphine utilization and discontinuation among individuals treated for opioid use disorder in a United States commercially insured population. J Subst Abuse Treat.

[33] American Psychiatric Association. Diagnostic and statistical manual of mental disorders (5th ed.). 2013. 10.1176/appi.books.9780890425596.

[34] Forder A (1976). Social work and system theory. Br J Soc Work.

[35] Ryan RM, Deci EL (2000). Self-determination theory and the facilitation of intrinsic motivation, social development, and well-being. Am Psychol.

[36] Perkins DD, Zimmerman MA (1995). Empowerment theory, research, and application. Am J Community Psychol.

[37] Elwyn G, Frosch D, Thomson R, Joseph-Williams N, Lloyd A, Kinnersley P (2012). Shared decision making: a model for clinical practice. J Gen Intern Med.

[38] Vaux A. Social support: theory, research, and intervention. Praeger Publishers; 1988. p. xiv, 346-xiv.

[39] Feldstein AC, Glasgow RE (2008). A Practical, Robust Implementation and Sustainability Model (PRISM) for integrating research findings into practice. Jt Comm J Qual Patient Saf.

[40] Miller CJ, Barnett ML, Baumann AA, Gutner CA, Wiltsey-Stirman S (2021). The FRAME-IS: a framework for documenting modifications to implementation strategies in healthcare. Implement Sci.

[41] U.S. Department of Health and Human Services NIoH, National Institute of Allergy and Infectious Diseases, Division of AIDS. Division of AIDS (DAIDS). Table for grading the severity of adult and pediatric adverse events, corrected version 2.1. 2017. Available from: https://rsc.niaid.nih.gov/sites/default/files/daidsgradingcorrectedv21.pdf. Cited 12-10-2023.

[42] ICMJE. Recommendations for the conduct, reporting, editing, and publication of scholarly work in medical journals. 2023. [updated May 202311/14/2023]. Available from: https://www.icmje.org/icmje-recommendations.pdf. 25558501

[43] Iyengar S, Kravietz A, Bartholomew TS, Forrest D, Tookes HE (2019). Baseline differences in characteristics and risk behaviors among people who inject drugs by syringe exchange program modality: an analysis of the Miami IDEA syringe exchange. Harm Reduct J.

[44] Allen ST, Ruiz MS, Jones J (2016). Assessing syringe exchange program access among persons who inject drugs (PWID) in the district of Columbia. J Urban Health.

[45] Samji H, Cescon A, Hogg RS, Modur SP, Althoff KN, Buchacz K (2013). Closing the gap: increases in life expectancy among treated HIV-positive individuals in the United States and Canada. PLoS One.

[46] Antiretroviral Therapy Cohort Collaboration (2008). Life expectancy of individuals on combination antiretroviral therapy in high-income countries: a collaborative analysis of 14 cohort studies. Lancet.

[47] Metzger DS, Woody GE, McLellan AT, O’Brien CP, Druley P, Navaline H (1993). Human immunodeficiency virus seroconversion among intravenous drug users in- and out-of-treatment: an 18-month prospective follow-up. J Acquir Immune Defic Syndr (1988).

