# Supplementary material for: Delivering integrated strategies from a mobile unit to address the intertwining epidemics of HIV and addiction in people who inject drugs: the HPTN 094 randomized controlled trial protocol (the INTEGRA Study)
Source: Trials. 2024 Feb 15;25:124. doi: 10.1186/s13063-023-07899-5 (PMC10870682; doi:10.1186/s13063-023-07899-5)
Supplement: Supplementary file 1 — Additional file 1. Sample informed consent form. [file 13063_2023_7899_MOESM1_ESM.pdf]

SAMPLE INFORMED CONSENT FORM- MAIN STUDY

HPTN 094
INTEGRA: A Vanguard Study of Health Service Delivery in a Mobile Health Delivery Unit to Link Persons who Inject Drugs to Integrated Care and Prevention for Addiction, HIV, HCV and Primary Care

Version 2.0
20February 2023
DAIDS Document ID: 38715

Sponsored by: Division of AIDS, US National Institute of Allergy and Infectious Diseases, US National Institutes of Health.

PRINCIPAL INVESTIGATOR: *[Insert Name*]

PHONE: *[Insert Number]*

<u>Key Information</u>:

The first two pages of this document include summary information about this study that will help you decide whether or not you should participate. More detailed information is provided after this summary section.

About this research
You are being asked to join a research study. Scientists do research to answer important questions which might help change or improve the way we do things in the future. This form explains the research study and your part in the study. Please read it carefully and take as much time as you need. Ask your study doctor or the study team to explain any words or information that you do not understand. You may take this description home and discuss it with your family or friends to help you decide.

Taking part in this research study is voluntary
You may choose not to take part in the study or may choose to leave the study at any time. Deciding not to participate, or deciding to leave the study later, will not result in any penalty or loss of benefits to which you are entitled and will not affect your relationship with the study site.

Important Information
This information gives you an overview of the research. More information about these topics may be found in the pages that follow.

1. **Why is this research being done?**
   This research study will help us see whether providing medical services in a mobile unit will help people who inject drugs (PWID) start and stay on anti-HIV medicines and medicines for opioid use disorder better.

2. **What will happen to me during the study?**
You will either receive health care services at visits conducted from a mobile unit or you will be guided to regular clinics in the area for your health care. All participants at Screening and Enrollment will receive laboratory testing to evaluate opiate use, HIV, hepatitis and sexually transmitted diseases. Participants will also receive a physical exam, counseling and develop a clinical plan with the health care providers. All participants will be assigned a peer health navigator to work with them during the study.

3. **How long will I participate in the study?**
If you decide to join the study, participation will last about 1 year with two visits after enrollment.

4. **Will I benefit from the study?**
It is possible that you may benefit from taking part in this study; however, there is no guarantee that it will help you. You will get information about your health and the results of the tests, as well as treatment for sexually transmitted infections. The counseling you get during this study may help you avoid HIV and other sexually transmitted infections. For more information, please see below.

5. **Will taking part in the study expose me to risks?**
Taking part in this research may expose you to risks. We may not know or understand all the risks at this time. It is very important that you understand the risks in this research study before you decide whether you will participate. For details and a list of risks you should know about, please see below.

6. **Will I be paid to participate?**
Payment for your time or travel is available if you decide that you will take part in this study. For more information, please see below.

7. **Will it cost me anything to participate?**
There is no cost to you for taking part in this study.

Please review the rest of this document for details about these topics and additional things you should know before making a decision about whether you will participate in this research.

INTRODUCTION

You are being asked to take part in a research study for people who inject drugs (PWID) with opioid use disorder (OUD) who are either without human immunodeficiency virus (HIV) or who are living with HIV which means you may either be at risk for getting HIV or could pass HIV on to others. HIV is the virus which causes acquired immunodeficiency syndrome (AIDS). This research study will help us see whether providing medical services in a mobile unit will help PWID start and stay on anti-HIV medicines and medicines for opioid use disorder better.

Before you decide whether to join the study, we will explain the purpose of the study, the risks and benefits to you, and what is expected of you if you decide to participate.

A research team member will talk with you about the study, tell you what will happen if you decide to participate in this study and give you this consent form to read. If you do not understand what you are reading please ask the study staff to explain anything you do not understand, including any language contained in this form. You may ask to have this form read to you.

After the study is explained to you and all your questions have been answered, you can decide whether or not you want to join the study. You do not have to make a decision now; you can take the consent document home and share it with family, friends, and your health care provider. Taking part in the study is voluntary which means you can choose whether or not to participate. If you join the study, you can then choose to leave at any time and it will not affect your eligibility to receive regularly-provided health services.

If you decide to join the study, you will be asked to sign this consent form and a copy of this form will be given to you. Keep this form; in it you will find contact information for the research staff and answers to questions about the study. If you choose not to take a copy of this form, we will give you a card with the research staff contact information.

1. **You should know key information about this study before you join.**

Here is a summary of important information about the study:

- This is a research study. Research is not the same as routine treatment or medical care. The purpose of a research study is to answer scientific questions. These answers can help find better ways to deliver treatment and improve knowledge of human behavior.
- Your participation in this study is voluntary. You may refuse to join, or you may withdraw your consent to be in the study, at any time, for any reason.
- We are doing this study to see whether providing medical services in a mobile unit will help PWID start and stay on opioid use treatment medications and anti-HIV medications.
- About 450 PWID either without HIV or who are living with HIV will participate in this study from cities in the United States. Participants will be in the study for about one year.
- We will collect blood, urine, and swabs from you at study visits to test for sexually transmitted infections (STIs). Collecting blood samples may cause pain, bruise your arm or make you feel lightheaded.
- We will test you for HIV, hepatitis A, B, and C and STIs during the study. You will receive treatment or referral for treatment as appropriate. The STIs we will test for are Chlamydia, gonorrhea, and syphilis. There are other STIs, but we will not test for them as part of this study.
- Before a study visit or at the start of a visit, we will assess you for COVID-19. If you are suspected to have COVID-19, you will need to wait until it is safe for you to be seen by study staff before we can conduct the visit.
- You may be provided medication for opioid use disorder (MOUD) and anti-HIV medications for HIV treatment or prevention, or you will be referred to places where you can get these medications.
- There may be some social risks. You may feel embarrassed or uncomfortable with some of the questions you will be asked, some of the procedures that will be done, or some of the test results that you will receive.
- We will make every effort to protect your confidentiality during the study. However, it is possible that others may learn that you are part of this study.
- The counseling you receive during this study may help you to avoid HIV, hepatitis A, B, and C and other STIs.
- If you join this study you will receive many health services at no charge while you are participating in the research. As well, the information gathered during this study may help to develop ways to prevent HIV and other STIs and to increase use of treatment for OUD among PWID and their partners in your community.

More information is given in this form about the study. You should feel that you understand the study before deciding whether you will participate.

ABOUT THE STUDY

The HIV Prevention Trials Network (HPTN) and *[insert site name]* are doing this study to see whether providing medical services in a mobile unit will help PWID start and stay on medications. The medications we mean here are for treatment of OUD as well as for treatment or prevention of HIV. About 450 PWID will participate in this study from cities in the United States. Participants will be in the study for about one year.

2. The study is testing a program to provide care for HIV and opioid use disorder to PWID in a mobile health delivery unit ("mobile unit").

Opioid use is the leading cause of accidental death in the United States. Injecting drugs can put people at high risk for getting HIV or passing HIV on to others. It can be difficult for people who inject opioids to start or stay on medication treatment offered at clinics. These clinics can be hard to get to and sometimes people have to go to multiple clinics to get all the services they need. We want to see whether providing multiple services in one location—the mobile unit—in a convenient place will make it easier to get treatment for OUD and HIV prevention and treatment. Medications offered in the mobile unit will be the same medications used in the regular clinics in this city. The only difference will be that they are provided in the mobile unit. This study may show a new way to improve the health of PWID.

3. Participants will be placed in 1 of 2 groups.

All participants will be assigned to one of two groups by random chance (the equivalent of flipping a coin). The difference between the groups is that the first group will receive health care services delivered on a mobile unit. These services include MOUD, HIV testing, HIV treatment for people living with HIV and not in care, pre-exposure prophylaxis (PrEP) for people who do not have HIV, and STI testing and treatment. The second group will have their initial Screening and Enrollment Visits in the mobile unit, but all other visits will occur in regular clinics in the area.

Participants in both groups will be assigned a peer navigator for 26 weeks who will assist participants in accessing and remaining in medical care.

All participants will come in for study assessments at approximately six and 12 months after starting the study and will be in the study for about a year total.

JOINING THE STUDY

4. It is your decision whether to participate in the study.

This consent form gives information about the study that will be discussed with you. We will help you understand the form and answer your questions before you sign this form. Once you understand the study, and if you agree to take part, you will be asked to sign your name or make your mark on this form. You will be offered a copy of this form to keep.

Before you learn about the study, it is important that you know the following:

- Your participation is voluntary. You do not have to take part in any of the tests or procedures in the study.

- You may decide not to take part in the study, or you may decide to leave the study at any time. Your decision will not affect your eligibility for continuing to receive health care at community-based services.
- If you decide not to take part in the study, you can still join another study at a later time if there is one available and you qualify.
- You cannot join this study if you are taking part in another research study, without special permission. You are asked to tell the study staff about any other studies you are taking part in or thinking of taking part in.

5. You must qualify before you can join the study.

If you decide to join this study, we will first do some tests and collect some information from you to find out if you qualify. These tests and the information collected are described in #6 below. If you do not qualify, you cannot join the study.

6. We will ask you questions, examine you, and test your blood.

To find out if you qualify, we will first conduct a "Screening Visit." Your Screening Visit will happen after you read, discuss, understand, and sign this form. The Screening Visit will take about X hours [*sites to fill in the amount of time]*.

At the Screening Visit, we will:

- Ask you about symptoms of COVID-19 and take your temperature.
- Ask you some questions about yourself, like your age, and your ethnic group.
- Ask where you live and how to contact you
- Provide drug use and HIV risk reduction counseling
- Collect ~*XX* mL (about *x* teaspoons) [*sites to complete*] of blood for HIV testing
- Collect urine to test for drug use and drug treatment
- Ask you questions about your sexual and drug use behaviors and drug use history including an assessment for Opioid Use Disorder (OUD) and recent injection drug use (track marks)
- Offer you condoms and lubricant and counsel you on how to use them safely
- Provide you with unused needles and dispose of any used needles you have [*sites to include if they are allowed to provide in their location*]
- Offer you a naloxone kit that can be used to treat an overdose, and show you how to use it

We may assess you before or at the start of the visit for COVID-19. If you are suspected to have COVID-19, you will need to wait until it is safe for you to be seen by study staff before continuing with screening for the study.

The initial results of the HIV test will be available immediately. If your initial test results indicate that you have HIV, we will perform additional testing in a laboratory to confirm these results and these will be available [*site to insert timeframe of testing*]. A small amount of blood will be stored from this visit. No other samples collected at the time of screening will be kept or used for any other tests other than those listed above.

7. **We will confirm if you qualify for the study.**

If the information you provide and the test results from the Screening Visit indicate that you may be eligible for the study, you will be asked to return for an "Enrollment Visit". This visit will last about X hours [*sites to fill in the amount of time]*.

During the Enrollment Visit, we will first determine if you are eligible to enroll in the study. If you are enrolled, you will complete additional activities. We expect all Enrollment Visit activities to be completed on the same day. If they cannot be completed on the same day, you will need to come back to finish the procedures on another day.
At the Enrollment Visit, before you're enrolled in the study, we will:

- Ask you questions to assess for COVID-19 infection and take your temperature. If you are suspected to have COVID-19, you will need to wait until it is safe for you to be seen by study staff before continuing with the visit.
- Confirm where you live and how to contact you.
- Collect urine to test for drug use and drug treatment, STIs,, and, if you are enrolled, to check for pregnancy (if you are someone who can become pregnant)
- Collect ~*XX* mL (about *x* teaspoons) [*sites to complete*] of blood for HIV testing and STIs. If you are enrolled, we will then also test your blood for hepatitis (a liver disease) and to assess the health of your blood and liver.
- Provide you with HIV test results and HIV counseling.
- Ask you about your sexual practices and medical history, about your history of drug use and drug treatment.. We will also ask you about other research studies you are participating in.
- We will ask you to show us the places on your body where you've most recently injected drugs.
- Ask you questions about your medical history including MOUD treatment, HIV risk behaviors, and participation in other research studies. We will also ask you about your history of being in jail or prison, and about experiences of depression, anxiety or trauma, and your use of tobacco, alcohol and other drugs.
- Collect swabs from you to test for STIs. These swabs may be taken from the throat, rectum and vagina.
- Give you a physical exam, that includes measuring your height, weight, heart rate, temperature and blood pressure; looking into your mouth and throat; listening to your heart and lungs, feeling your neck and abdomen, looking at your skin, arms and legs, additional procedures if indicated for your care and asking you about any medicines you are taking.
- Give you the results of tests that are available during the visit and discuss your health needs.

8. **If you qualify, you will enter the study**

If you are eligible to participate in the study, you will be enrolled during the Enrollment Visit. You will then be placed by random chance into either the group that will receive medical care in the mobile health delivery unit, or the group that will not. After that, the Enrollment Visit will continue and we will:

- participationadditionalcareStore blood and urine samples for other testing related to this study. This may include tests for drugs, medications used to treat HIV, and medications to treat substance use. Tests may also be performed to characterize HIV and the body's response to HIV. Similar testing may be performed for hepatitis viruses. The stored blood samples may also be used to learn more about how HIV is spread throughout the community. Blood samples will also be used to test for possible exposure to the virus that causes COVID-19 and to learn more about how the body responds to COVID-19. Results from testing using stored samples will not be returned to you or the study site.

- Introduce you to a peer navigator, someone who has been trained to help you get medical services and be successful in getting treatment for OUD.
- If you have symptoms of an STI at the time of the visit, we will provide you with treatment right away. If laboratory testing of the samples collected at this visit show you have an STI, we will let you know, however these results will not be available until after this visit.
- Make a plan for treating your opioid use disorder and for starting anti-HIV medications to treat or prevent HIV.
- Offer you condoms and lubricant and counsel you on how to use them safely.
- Provide you with unused needles and dispose of any used needles you have. [sites to include if they are allowed to provide in their location]
- Offer you a naloxone kit that can be used to treat an overdose and show you how to use it

BEING IN THE STUDY

9. **You will have two study visits over a year. In addition to this you will work with your peer navigator several times over the first six months of the study. If you are in the group that will receive medical care in the mobile unit, you will also have medical visits in the mobile unit at various times over the first six months of the study.**

Study Visits
If you join the study you will have at least two additional study visits after your Enrollment Visit. These visits will be approximately six months (26 weeks) and 12 months (52 weeks) after the Enrollment Visit. Participants who become incarcerated during the Study will be scheduled for study activities for which they are eligible upon release.

During the **week 26 and 52 study visits**, we will:

- Ask you questions to assess for COVID-19 infection and take your temperature. If you are suspected to have COVID-19, you will need to wait until it is safe for you to be seen by study staff before continuing with the visit.
- Confirm where you live and how to contact you.
- Ask you questions about your sexual practices and medical history, about your history of drug use and drug treatment, about your history of being in jail or prison, and about experiences of depression, anxiety or trauma.
- Talk with you about HIV and ways to protect yourself from getting it if you do not have HIV.
- Collect ~*XX* mL (about *x* teaspoons) [*sites to complete*] of blood for HIV testing if you are not already known to be living with HIV and to test your blood for STIs and hepatitis (a liver disease). We will test the level of HIV virus in your blood (viral load) if you have HIV.
- Collect urine to test for drug use and drug treatment, and to check for pregnancy (if you are someone who can become pregnant)
- Collect swabs from you to test for STIs. These swabs may be taken from the throat, rectum and vagina.
- We will store blood and urine samples for other testing related to this study. This may include testing for drugs, medications used to treat HIV and substance use, and other tests related to HIV and HCV infection. The stored blood samples may also be used to learn more about how HIV and HCV are spread throughout the community. Blood samples will also be used to test for possible exposure to the virus that causes COVID-19 and to learn more about how the body responds to COVID-19. Results from testing using stored samples will not be returned to you or the study site.
- Provide treatment if you have symptoms of an STI.

- Give you the results of tests when they are available.
- If your laboratory testing shows that you have an STI, we will refer you for treatment.
- Offer you condoms and lubricant and counsel you on how to use them safely.
- Provide you with unused needles and dispose of any used needles you have. [*sites to include if they are allowed to provide in their location*]
- Offer you a naloxone kit that can be used to treat an overdose and show you how to use it

Each of these visits will last about X hours [*site staff to insert amount of time]*. At your final study visit (week 52 visit), we will talk with you about what will happen when the study is over, including when the results of the study will be available.

Visits with Your Peer navigator
You will work with a peer navigator during the first six months of the study. Your peer navigator will help you address issues that may have made it hard for you to get care in the past. They will act as a coach to help you stay motivated in treatment for opioid use disorder and HIV care or prevention. They will remind you of medical appointments you have and help you find medical and other resources available to you in your community.

You will mostly work with your navigator in-person at the start of the six months. Over time you may work together more over the phone/through messaging apps. If you are in the group that receives medical care in the mobile unit, your navigator will help you transition your medical care to a clinic or clinics in your community before the six months is over. If you are in the group that does not get medical care in the mobile unit, your navigator will work with you to get medical care at a clinic or clinics in your community right from the start.

How often you interact with your navigator and how long these interactions last will depend on your needs. We expect you will be in contact with your navigator multiple times per month.

Medical Care Visits in the Mobile Unit
If you are put in the group that will receive medical care in the mobile unit, you will have multiple visits to the mobile unit over the first six months of the study. How often you have visits and how long they last will depend on your medical needs. This may change over time.

Note that if you already receive medical care from providers in the community and are happy with this care, you do not have to switch to care in the mobile unit. You can also choose to get some care in the mobile unit and other care from regular clinics in the community.

A primary goal of the care you receive in the mobile unit will be to provide you with medical treatment for opioid use disorder. The medications expected to be used in this Study will be buprenorphine or combination buprenorphine/naloxone. The Study Team will work with the U.S. Drug Enforcement Administration at national and local levels to comply with policies for dispensation of buprenorphine and buprenorphine/naloxone to participants. Another primary goal is to provide you with HIV treatment (ART) if you are living with HIV or to provide you with HIV prevention (PrEP) if you are not living with HIV.

What happens at a medical care visit will depend on your medical needs at the time. We would expect that they would include at least some of the following:

- Ask you questions to assess for COVID-19 infection and take your temperature. If you are suspected to have COVID-19, you will need to wait until it is safe for you to be seen by study staff before continuing with the visit.
- Confirm where you live and how to contact you.
- Ask you questions about your sexual practices, medical history, drug use and drug treatment history and how you feel about how your life is going.
- Talk with you about HIV and ways to protect yourself from getting it if you do not have HIV
- Give you a physical exam that may include measuring your weight, temperature, blood pressure, looking into your mouth and throat, listening to your heart and lungs, feeling your abdomen (stomach and liver), additional procedures if indicated for your care and asking you about any medicines you are taking.
- Begin HIV treatment if you are living with HIV. Offer PrEP if you are without HIV.
- Provide medications to treat opioid use disorder.
- Provide referral for vaccinations for hepatitis A and B.
- Provide referral for treatment, if you have hepatitis B or C.
- Collect ~*XX* mL (about *x* teaspoons) [*sites to complete*] of blood for HIV testing if you are not already known to be living with HIV and to test your blood for STIs. We will test the level of HIV virus in your blood (viral load) and the health of your blood (CD4 cell count) if you have HIV.
- Collect urine to test for drug use, and to check for pregnancy (if you are someone who can become pregnant).
- Collect swabs from you to test for STIs. These swabs may be taken from the throat, rectum and vagina.
- We will store blood samples for other testing related to this study at any visit where HIV testing is done. This may include testing for medications used to treat HIV and other tests related to HIV and HCV infection. The stored blood samples may also be used to learn more about how HIV and HCV are spread throughout the community. Results from testing using stored samples will not be returned to you or the study site.
- Provide treatment if you have symptoms or laboratory results indicating an STI.
- Give you the results of tests when they are available.
- Offer you condoms and counsel you on how to use them safely.
- [*Sites to include if allowed at their location*: “Provide you with unused needles and dispose of any used needles you have.”]
- Offer you a naloxone kit that can be used to treat an overdose and show you how to use it

10. If you stop participating in peer navigation, or stop receiving OUD or HIV care or prevention, we will ask you to stay in the study.

We hope that if you join this study, you will work with your peer navigator for the full six months. We also hope that you will start and stay on treatment for OUD and receive HIV treatment or prevention for the full six months (in the mobile unit if that is your group). But if you stop these activities for any reason, we still ask you to continue to come for the study visits at 26 and 52 weeks. The data you provide at these study visits will still be important to the outcome of this study. You always have the right to stop participating in the study completely at any time.

11. If you acquire HIV during the study, we will help you get care and support.

We will test your blood for HIV during this study. If you are without HIV when you join the study and test positive for HIV while you are in the study you will stop taking PrEP if you started PrEP. You will be asked to come back to provide an additional ~*XX* mL (about *x* teaspoons) [*sites to complete*] blood to

confirm the HIV result and to provide additional samples for other assessments. You will also be asked to complete the remaining study visits (26 and/or 52 weeks). We will provide or help you find the care and support you need. You will still continue to receive peer navigation through six months and will continue to receive medical care in the mobile unit, if you are in the group that receives care in the mobile unit.

12. **We will test you for pregnancy during the study if you are someone who can become pregnant.**

We will collect urine to test for pregnancy and will give you these results each time. If you become pregnant during the study, you will be able to stay in the study as originally planned. If you are pregnant at the last study visit, we will contact you after that to find out the outcome of the pregnancy.

Even if you are in the group to receive medical care in the mobile unit, the study cannot provide all the medical care you will need for a pregnancy or delivery of a baby. The baby will not be in the study. Therefore, it is important to receive medical care for a pregnancy outside the study. We will tell you where you can go for the care you need.

If you are taking ART or PrEP and become pregnant, we will provide some basic information about your pregnancy, such as what medications you took while pregnant, what prenatal care you received, and the outcome of your pregnancy, to the Antiretroviral Pregnancy Registry. None of your personal information, such as your name or address, will be provided to the registry. You can learn more about this registry at www.apregistry.com.

13. **Some of your blood and urine may be left over at the end of the study and may be used for future research, if you provide consent.**

Some of your blood and urine collected for this study may be left over after all of the study tests are completed. If you agree, these stored samples may also be used for future research related to HIV infection, hepatitis infection, COVID-19, and other infections, and to better understand laboratory tests related to this study. This research will not include whole genome sequencing also known as WGS.

You will be asked to sign this consent form to give permission to use your stored samples for future research. Even if you do not give permission to store your blood for possible future research, you can still be in this study. If you give permission, you will not be asked to give permission again during the study. However, you may withdraw your consent to use your stored samples for future research at any time. We will then destroy your samples after all of the study-related testing has been completed. If you agree to have your stored samples used for future research, there is no time limit on how long your samples will be stored. The stored samples will be labeled only with your study number and will be tested at the HPTN Laboratory Center (LC) or laboratories designated by the HPTN LC. We will not share the key that says which study number is yours so the laboratory doing the testing will not know who you are. Only approved researchers will have access to your samples. Results from this testing will not be returned to the study site or you. Your samples will not be sold or directly used to produce commercial products or for commercial gain. All proposed research studies using your samples will be reviewed by the National Institutes of Health (NIH).

RISKS OF THE STUDY

14. **There may be risks to being in this study.**

STUDY PROCEDURES

Taking blood samples may cause some pain, bruise your arm, or make you feel lightheaded. In rare cases you may faint. There is also a slight chance of infection when blood is drawn. You may be nervous while you are waiting for your HIV or other test results. If the tests show that you have HIV or another infection, you may worry about your health and future. You will receive counseling before and after the test to help address your concerns.

You may experience pain or discomfort in your throat, rectum or vagina from the swab. In some cases, you may have some bleeding.

You will be tested for gonorrhea, chlamydia, syphilis, and hepatitis. [*Note to sites: Insert here any reporting responsibilities for your state or local jurisdictions or reporting of these infections to public health authorities. Also include whether if a participant tests positive, the results will become part of public health records, or any other record (medical file, etc.)].*

DISCLOSURE OF PERSONAL INFORMATION

We will make every effort to protect your confidentiality during the study. However, it is possible that others may learn that you are part of this study and they may think that you are living with HIV or are at high risk for acquiring HIV. They could make assumptions about your use of drugs. Because of this, you could face stigma related to HIV or drug use. This could cause you trouble finding or keeping a job. You could also have problems with your family, friends and community. If you work with a peer navigator, you may choose to communicate with your navigator by text or messaging apps. If others have access to your phone, they may be able to see these communications. You should consider carefully what kind of information you want to share in this way. We can tell you more about how we will protect your information.

SENSITIVE QUESTIONS

The questions we will ask you like about your sexual practices, medical history, drug use, history of being in jail or prison, experiences of depression, anxiety or trauma may make you feel uneasy. However, you do not have to answer any question that you do not want to and you can stop answering the questions at any time.

SIDE EFFECTS OF MEDICATIONS

If you are assigned to the group that **will NOT receive medical care in the mobile health delivery unit**, you may receive medications from the study staff at the Enrollment Visit. These will be medications to treat STIs, if you have STI symptoms at the Enrollment Visit. The clinician will explain to you any side effects associated with those medications. After the Enrollment Visit, your peer navigator will help you receive care at the regularly available clinics in your community. The staff at those facilities can explain to you the side effects of any medications they give to you or prescribe for you. You will also be offered naloxone kits during your study participation that can be used to treat an overdose. The side effects of naloxone will be explained to you.

If you are assigned to the group that **WILL receive medical care in the mobile unit**, you will receive or be prescribed medications by the study staff during the first six months of your participation in the study. The drugs you could be given on the **mobile unit** include medications to treat or prevent STIs or other bacterial infections, hepatitis, HIV, opioid use disorder or overdose. You may also be prescribed medications that you will need to pick up at a pharmacy. The clinician will explain to you the side effects of the specific medications you are prescribed or given.

IF YOU ARE PREGNANT OR BECOME PREGNANT WHILE PARTICIPATING IN THE STUDY
The medications provided in this study are the same types of drugs that would be provided as part of regular care at clinics in your community and are commonly provided to pregnant people. There is no greater risk to you by receiving them as part of this study. If you are in the group that receives medical care in the **mobile unit**, your clinician will tell you if the medications you are taking have any risks or side effects that apply to pregnant people. If you are in the group that does not receive medical care in the **mobile unit**, you should tell your clinician that you are pregnant so that they can explain to you any risks or side effects of your treatment that apply to pregnant people.

BENEFITS OF THE STUDY

15. There may be direct and indirect benefits to you from participating in the study.

If you participate in this study, you will receive many health services at no charge. You will be tested for HIV, hepatitis A, B, and C, and other STIs. You will receive treatment or referral for treatment, as appropriate, if you have these infections. The counseling you get during this study may help you to avoid HIV, hepatitis A, B, and C, and other STIs. If you have or get HIV, this counseling may help you to learn how to better care for yourself and avoid passing HIV to your sexual partners (or fetus, if you are or become pregnant). If you do not have HIV, you will be offered PrEP or referral for PrEP to help you avoid getting HIV. Your peer navigator will work to link you to health services, including treatment for OUD and HIV care or prevention services. You will receive free condoms and lubricant, and naloxone kits for overdose reversal. If you are put in the group that receives medical services in the mobile unit, you will be provided with medical care and medication in a single convenient location.

If you are pregnant or become pregnant while participating in the study, there may be benefits to your fetus from participating in this study. Being on medical treatment for OUD may let you take better care of yourself, which will benefit your fetus. Taking OUD medication may decrease your use of street drugs, which can also benefit your fetus. Taking medication to either treat or prevent HIV while you are pregnant can help you prevent passing HIV on to your fetus.

You may receive indirect benefits from this study. The information gathered during this study may show how medical services can be provided more successfully to people who inject drugs, particularly services for OUD and HIV. This could influence how medical services are provided in the future and may be beneficial to you and your community.

OTHER INFORMATION ABOUT THE STUDY

16. Some of the information we collect from you for this study may be used for other research.

Information collected from this study may be used for other studies carried out by this team or by other researchers. The information we share with other researchers would never include your name or any other information that could identify you.

17. We will tell you any new information that may affect your decision to be in the study.

You will be told any new information learned during this study that might affect your willingness to stay in the study. You will also be told when the results of the study may be available, and how to learn about them.

18. You may be withdrawn from the study without your consent.

We may take you out of the study at any time without your consent. This may happen if:

- You are unable or unwilling to follow all of the study procedures or instructions.
- The study is stopped or canceled.
- The study staff feels that staying in the study would be harmful to you.
- Other reasons, as decided by the study staff.

If we take you out of the study, we may ask you to come back to the clinic one last time to collect a blood sample and ask you questions.

19. You have other choices if you choose not to be in this study.

[*Sites to include/amend the following if applicable]* There may be other studies going on here or in the community that you may be eligible for. If you wish, we will tell you about other studies that we know about. There also may be other places where you can go for HIV counseling and testing, HIV treatment or PrEP, and medication for OUD. We will tell you about those places if you wish.

20. There is no cost to you to be in this study.

There will be no cost to you for study related visits, physical examinations, laboratory tests, or other procedures or medications you receive in the mobile health unit.

The study will not pay for any medications or health services you receive outside of the mobile unit. This is true even if you are given a prescription or a referral for these by the staff in the mobile unit. However, study staff will help connect you to health services in the community that may be free or low-cost and will help you to sign up for health insurance that you are eligible for.

21. We will give you [site to insert amount] for each study visit.

You will receive [$xx] for your time, effort, and travel to and from the clinic at each scheduled visit. We may invite you to refer people you know to enroll in this study and offer to pay you if those people enroll. If so, you will receive [$xx] for each of these referrals. [*Sites to insert information about local reimbursement for the study.*]

22. We will do our best to protect your private information.

Every effort will be made to keep your personal information confidential, but we cannot guarantee absolute confidentiality. To keep your information private, the samples of your blood and urine that are used for research purposes will be labeled with a code that can only be traced back to your study clinic. Your name, where you live, and other personal information will be protected by the study clinic. The results of any tests done on these research samples will not be included in your health records. You will be identified by a code, and personal information from your records will not be released without your written permission. Any publication of this study will not use your name or identify you personally. Your personal information may be disclosed if required by law.

Clinic staff will have access to your study records. Your records may also be reviewed, under guidelines of the US Federal Privacy Act, by:

- The [*insert name(s) of Single Institutional Review Board (IRB)]*
- The sponsor of the study (US National Institutes of Health [NIH]), its contractors, and its study monitors
- the National Institute on Drug Abuse (NIDA) and/or their contractors
- The US Office for Human Research Protections (OHRP)
- other local, US, and international regulatory entities
- Personnel of the HPTN research network that is conducting this study
- And (*insert any other applicable local authorities*]

In addition to the efforts made by the study staff to help keep your personal information confidential, we have obtained a Certificate of Confidentiality from the US Federal Government. This Certificate protects researchers from being forced to tell people who are not connected with this study, such as the court system, about your participation. The Certificate of Confidentiality does not prevent you from releasing information about yourself and your participation in the study. The Certificate cannot be used to resist a demand for information from personnel of the US Government that is used for auditing or evaluation of federally funded projects or for information that must be disclosed in order to meet the requirements of the US FDA. Even with the Certificate of Confidentiality, if the study staff learns of possible child abuse and/or neglect or a risk of harm to you or others, we will tell the proper authorities.

The study staff will also use your personal information, if needed, to verify that you are not taking part in any other research studies. This includes other studies conducted by [*site name*] and studies conducted by other researchers that study staff know about.

[Sites to include/amend the following if applicable:] [*Local/state/national*] regulations require study staff to report the names of people who test positive for [*HIV and other infections*] passed during sex to the [*local health authority*]. Outreach workers from the [*health authority*] may then contact you about informing your partners, since they also should be tested. If you do not want to inform your partners yourself, the outreach workers will contact them, according to the confidentiality guidelines of the [*health authority*].

The virus in one person with HIV is slightly different from the viruses in other people. If you give us permission to save your samples for future testing, and you are living with HIV, we may use special laboratory tests to compare the virus in your blood to the virus from other people with HIV. This can help us understand how HIV is spread throughout communities. The samples used for this testing will not include any information that would identify you, like your name. This is to prevent anyone from being able to connect any information we learn about your HIV to you. The results of this analysis will only be used for research.

23. If you get sick or injured during the study, contact us immediately.

[Sites to specify institutional policy:] It is unlikely that you will be injured as a result of study participation. If you are injured, the [*institution*] will give you immediate necessary treatment for your injuries. You [*will/will not*] have to pay for this treatment. You will be told where you can get additional treatment for your injuries. There is no program to pay money or give other forms of compensation for such injuries either through this institution or the US NIH. You do not give up any legal rights by signing this consent form.

24. Contact us at any time if you have questions or problems.

If you ever have any questions about the study, or if you have a research-related injury, you should contact [*insert name of the investigator or other study staff*] at [*insert telephone number and/or physical address*].

An institutional review board (IRB) is an independent committee established to help protect the rights of research participants. If you have any questions about your rights as a research participant, and/or concerns or complaints regarding this research study, contact**:**

- By mail:
  Study Subject Adviser
  Advarra IRB
  6100 Merriweather Drive
  Suite 600
  Columbia, MD 21044
- or call **toll free**: 877-992-4724
- or by **email**: adviser@advarra.com

If you have questions about who to contact at the research site, you should contact [insert name of the investigator or community educator or CAB member] at [insert physical address and telephone number]

SIGNATURE PAGE

HPTN 094
INTEGRA: A Vanguard Study of Health Service Delivery in a Mobile Health Delivery Unit to Link Persons who Inject Drugs to Integrated Care and Prevention for Addiction, HIV, HCV and Primary Care

Version 2.0
20 February 2023

(Modify as needed per protocol requirements)
Insert signature blocks as required by the local IRB:] If you have read this consent form, or had it read and explained to you, and you understand the information, and you voluntarily agree to join the study, please sign your name or make your mark below. Also, please indicate by providing your initials in the spaces below if you agree to long-term storage and future testing of your samples.

________ I agree to take part in this study.

________ I agree to have samples of my blood stored for future testing related to HIV infection, HCV infection, COVID-19, other infections, and other goals of this study.

________ I agree to have samples of my urine stored for future testing related to HIV infection, HCV infection, COVID-19, other infections, and other goals of this study

________ I do not agree to have samples of my blood stored and used for future testing.

________ I do not agree to have samples of my urine stored and used for future testing.

____________________ ____________________________________
Participant Name (print) Participant Signature and Date

____________________ ____________________________________
Study Staff Conducting Consent Discussion (print) Study Staff Signature and Date

____________________ ____________________________________
Witness Name (print) (As appropriate) Witness Signature and Date

APPENDIX III: SAMPLE INFORMED CONSENT FORM- QUALITATIVE INTERVIEWS

HPTN 094
INTEGRA: A Vanguard Study of Health Service Delivery in a Mobile Health Delivery Unit to Link Persons who Inject Drugs to Integrated Care and Prevention for Addiction, HIV, HCV and Primary Care

Final Version2.0
20 February 2023
DAIDS Document ID: 38715

Sponsored by: Division of AIDS, US National Institute of Allergy and Infectious Diseases, US National Institutes of Health.

PRINCIPAL INVESTIGATOR: *[Insert Name]*

PHONE: *[Insert Number]*

KEY INFORMATION

- Participation in this research study is entirely voluntary.
- This study is part of a larger research project focused on people who inject drugs with opioid use disorder. That project is testing whether providing medical services in a mobile unit can help people who inject drugs stay on anti-HIV medicines and on medicines for opioid use disorder.
- This research study will involve conducting interviews with different kinds of people to better understand what makes it easier or harder to successfully offer medical services in a mobile unit to people who inject drugs. Your participation will consist of a one-time, audio-recorded interview, conducted in-person or via phone/internet, and is expected to last 30-60 minutes.
- Risks or discomforts associated with participating in this research study include potential feelings of discomfort due to the interview questions, as well as potential breaches of confidentiality and privacy.
- It is unlikely that you will receive any direct benefit from participating in this research study. We hope that the information gathered from these interviews lead to better health options in the future for people who inject drugs.

INTRODUCTION

You are being asked to participate in an interview to help us understand what makes it easier or harder to successfully offer medical services in a mobile unit to people who inject drugs. We will interview different kinds of people to answer this question, potentially including leaders in the community, health care providers, people who inject

drugs and people who work with people who inject drugs, law enforcement, harm reduction leaders and advocates, medical examiners/coroner's office, and scientists who are involved in studying PWID in the area.

VOLUNTARY PARTICIPATION

Your participation in this study is voluntary. You may refuse to join, or you may withdraw your consent to be in the study, at any time, for any reason. *[For potential interviewees who are participants in the main study: "*If you decide not to take part in this study, or if you join this study and then decide to leave, it will not alter the medical care you are eligible to receive. It also will not affect your ability to participate in the main study."*]* [*For potential interviewees who are staff members at a site*: "If you decide not to take part in this study, or if you join this study and then decide to leave, it will not have any effect on your employment. You have the same right to decide whether to participate as main study participants do and will not be viewed as "uncooperative" if you choose not to participate."] Although we hope that you will be comfortable answering all of the questions openly and honestly, please remember that you may refuse to answer any of the questions, or stop participating in the interview completely, at any time.

Before you decide whether to join the study, we would like to explain the purpose of the study, the risks and benefits to you, and what is expected of you.

This consent form gives information about the study that will be discussed with you. We will help you understand the form and answer your questions before you sign this form. Once you understand the study, and if you agree to take part, you will be asked to sign your name or make your mark on this form. You will be offered a copy of this form to keep.

PURPOSE OF THE STUDY

The purpose of this study is to learn what makes it easier or harder to successfully offer medical services in a mobile unit to people who inject drugs. The information you give in this interview will be combined with information collected during the main research study. We expect to interview approximately 195 people (39 people total in this city) from the cities where the main research study is being conducted. We hope that in the future the information collected in this study will allow medical care to better meet the needs of people who inject drugs.

STUDY PROCEDURES

If you decide to participate in this study, you will have one interview. The interview will be conducted by a member of the research team either in person or via phone. They will ask you where you live and how to contact you. They will ask you specific interview questions to address the overall question

[*for local public health officials: "what public health infrastructures would be needed to deliver medical services in a mobile unit to persons who inject drugs in the absence of this study?"]*
[*for staff at existing community-based services: "in what ways does delivery of medical services in a mobile unit affect how people who inject drugs are served in this community?"*]

[*for staff on the moble mobile unit: "what are the resource and personnel needs you have had to address to deliver medical services to people who inject drugs on a mobile unit?"*]
[*for study participants: "in what ways can having medical services in a mobile unit affect the health of people who inject drugs in this city?"*]

To help make sure that we fully understand your answers, the interviews will be audio-recorded. The information on the audio-recording will then be turned into a transcript (a written record of the conversation) by an individual who works with the research team. Your name will not be included in that written record.

- The interview requires only one study visit and **will take 30-60 minutes** to complete.
- There will be **no cost to you** to participate in the interview.
- **You will receive [*site to fill-in*]** for your time and effort.

RISKS AND/OR DISCOMFORTS

The risk to you in participating in this interview is that some of the questions may be uncomfortable or make you feel uneasy. However, you do not have to answer any question that you do not want to and you can stop answering the questions at any time. You will also be provided with contact and referral information if any of the questions raise issues that you would like to address at this or some later time. Other possible risks associated with this study may include breaches of confidentiality. Although study sites will make every effort to protect your privacy and confidentiality, it is possible that your involvement in the study could become known to others, and that social harms (e.g., unfair or discriminatory treatment) may result. To reduce the likelihood of these risks, all interviews will be conducted in a private setting, and names/identifying information will be removed from transcriptions to protect confidentiality.

BENEFITS

It is unlikely that you will receive any direct benefit from being in this study; however, information gathered during this study may lead to better health options in the future for people who inject drugs.

STUDY RESULTS

You will be told when the results of the study may be available, and how to learn about them.

WHY YOU MAY BE WITHDRAWN FROM THE STUDY WITHOUT YOUR CONSENT

You may be withdrawn from the study without your consent if any of the following occur:

You are unable or unwilling to follow all of the study procedures or instructions.

The study is stopped or canceled.

The study staff feels that staying in the study would be harmful to you.

You are not able to complete all study procedures.

Other reasons, as decided by the study staff.

CONFIDENTIALITY

The research team will protect your confidentiality by not putting your name on any audio files or interview transcripts. These items will be labeled with a code that can only be traced back to your study clinic and these items will be kept in a secure location that can only be accessed by the study staff. Your name and other personal information will be protected by the study clinic. Your information collected as part of this research study will not be used or distributed for future studies even if identifiers are removed.

Efforts will be made to keep your study records and test results confidential to the extent permitted by law. However, we cannot guarantee absolute confidentiality. Your personal information may be disclosed if required by law. You will be identified by a code, and personal information from your records will not be released without your written permission. Any publication of this study will not use your name or identify you personally. However, your records may be reviewed, under guidelines of the US Federal Privacy Act, by the sponsor of the study (US National Institutes of Health [NIH]) and/or its authorized representatives, the Institutional Review Board (IRB), study staff, study monitors, [*insert applicable local authorities*] and other local, US or international regulatory authorities.

In addition to the efforts made by the study staff to help keep your personal information confidential, we have obtained a Certificate of Confidentiality from the US Federal Government. This Certificate protects researchers from being forced to tell people who are not connected with this study, such as the court system, about your participation. The Certificate of Confidentiality does not prevent you from releasing information about yourself and your participation in the study. The Certificate cannot be used to resist a demand for information from personnel of the US Government that is used for auditing or evaluation of federally funded projects or for information that must be disclosed in order to meet the requirements of the US FDA. Even with the Certificate of Confidentiality, if the study staff learns of possible child abuse and/or neglect or a risk of harm to you or others, we will tell the proper authorities.

Your records may be reviewed by:

the US NIH

the US Department of Heath and Human Services (DHHS), Office for Human Research Protection (OHRP)

the National Institute on Drug Abuse (NIDA) and/or their contractors

[insert name of IRB]

US, local or international regulatory authorities/entities

study staff

study monitors

Other regulatory agencies

RESEARCH-RELATED INJURY

[Sites to specify institutional policy:] It is unlikely that you will be injured as a result of study participation. If you are injured, the [*institution*] will give you immediate necessary treatment for your injuries. You [*will/will not*] have to pay for this treatment. You will be told where you can get additional treatment for your injuries. There is no program to pay money or give other forms of compensation for such injuries either through this institution or the US NIH. You do not give up any legal rights by signing this consent form.

PROBLEMS OR QUESTIONS

If you have any questions about the study, or if you have a research-related injury, you should contact [*insert name of the investigator or other study staff*] at [*insert telephone number and/or physical address*].

If you have questions about your rights as a research participant, you should contact *[insert name or title of person on the IRB or other organization appropriate for the site]* at *[insert physical address and telephone number].*

If you have questions about who to contact at the research site, you should contact *[insert name of the investigator or community educator or CAB member]* at *[insert physical address and telephone number].*

SIGNATURE PAGE

HPTN 094
INTEGRA: A Vanguard Study of Health Service Delivery in a Mobile Health Delivery Unit to Link Persons who Inject Drugs to Integrated Care and Prevention for Addiction, HIV, HCV and Primary Care

Final Version 2.0
20 February 2023

If you have read this consent form, or had it read and explained to you, and you understand the information, and you voluntarily agree to join the study, please sign your name or make your mark below.

____________________	______________________________
Participant Name (print)	Participant Signature and Date
____________________	______________________________
Study Staff Conducting Consent Discussion (print)	Study Staff Signature and Date
____________________	______________________________
Witness Name (print) *(As appropriate)*	Witness Signature and Date
