# Supplementary material for: Delivering integrated strategies from a mobile unit to address the intertwining epidemics of HIV and addiction in people who inject drugs: the HPTN 094 randomized controlled trial protocol (the INTEGRA Study)
Source: Trials. 2024 Feb 15;25:124. doi: 10.1186/s13063-023-07899-5 (PMC10870682; doi:10.1186/s13063-023-07899-5)
Supplement: Supplementary file 2 — Additional file 2: Supplemental Table 1. WHO Trial Registration Data Set (Version 1.3.1). Supplemental Table 2. Footnotes for Table 2. Supplemental Table 3. Additional procedures for participants who have a reactive or positive HIV test after enrollment. [file 13063_2023_7899_MOESM2_ESM.docx]

**Supplemental Table 1. WHO Trial Registration Data Set (Version 1.3.1)**

| Element |  |
| --- | --- |
| Primary Registry and Trial Identifying Number | ClinicalTrials.gov, NCT04804072 |
| Date of Registration in Primary Registry | 2021-03-18 |
| Secondary Identifying Numbers | HPTN 094 |
| Source(s) of Monetary or Material Support | U.S. National Institute on Drug Abuse |
| Primary Sponsor | Division of AIDS (DAIDS), U.S. National Institute of Allergy and Infectious Diseases (NIAID), U.S. National Institutes of Health (NIH) |
| Secondary Sponsor(s) | None |
| Contact for Public Queries | Philip Andrew, RN,  919-544-7040,  pandrew@fhi360.org |
| Contact for Scientific Queries | pandrew@fhi360.org |
| Public Title | INTEGRA: A Vanguard Study of Health Service Delivery in a Mobile Health Delivery Unit |
| Scientific Title | INTEGRA: A Vanguard Study of Health Service Delivery in a Mobile Health Delivery Unit to Link Persons Who Inject Drugs to Integrated Care and Prevention for Addiction, HIV, HCV and Primary Care |
| Countries of Recruitment | United States |
| Health Condition(s) or Problem(s) Studied | Opioid use disorder, injection drug use |
| Intervention(s) | Experimental: Integrated health services delivered in the mobile unit and peer navigation. Participants in the intervention arm will be provided integrated health services delivered in the mobile unit and peer navigation for 26 weeks.  Active Comparator: Peer navigation to connect them to health services available at community-based agencies  Participants in the active control arm will be provided 26 weeks of peer navigation to connect them to health services available at community-based agencies. |
| Key Inclusion and Exclusion Criteria | Inclusion Criteria:   - At least 18 years of age - Urine test positive for recent opioid use and with evidence of recent injection drug use ("track marks") - Diagnosed with OUD per Diagnostic and Statistical Manual of Mental Disorders (DSM)-5 - Able and willing to give informed consent - Willing to start MOUD treatment - Able to successfully complete an Assessment of Understanding - Self-reported sharing injection equipment and/or condomless sex in the last three months with partners of HIV-positive or unknown status - Able to provide adequate locator information - Confirmed HIV status, as defined in the HPTN 094 Study Specific Procedures Manual   Exclusion Criteria:   - Urine testing that is not negative for methadone within 30 days prior to Enrollment is exclusionary, unless verified hospital records show methadone received as a medication for hospitalization only during the screening period. A volunteer may provide a sample for urine testing more than once during the screening period in order to achieve a negative result. If this criterion cannot be met within 30 days from the start of screening, the individual will be considered a screen failure and the volunteer has up to two more screening chances to successfully complete the screening process again. - Received MOUD in the 30 days prior to enrollment by self-report - Co-enrollment in any other interventional study unless approved by the Clinical Management Committee (CMC) |
| Study Type | Type:  Inteventional  Allocation:  Randomized  Interventional Model:  Parallel Assignment  Interventional Model Description:  Randomized, 1:1 study of 450 participants.  Masking:  None (Open Label)  Primary Purpose:  Prevention |
| Date of First Enrollment | 2021-06-02 |
| Sample Size | 447 (38 persons living with HIV) |
| Recruitment status | Complete |
| Primary Outcome(s) | Evaluate whether the intervention improves use of MOUD, as measured at 26 weeks, by assessing the following endpoint:   1. Alive 2. Retained 3. Biological evidence of MOUD (any detectable medications) 4. A MOUD prescription current at the Week 26 visit or proof of current receipt of MOUD from a clinic that does not provide individual MOUD prescriptions (e.g., methadone clinics)   Evaluate whether the intervention increases use of PrEP among people without HIV, as measured at 26 weeks, by assessing the following endpoint:   1. Alive, 2. Retained, 3. Without HIV, 4. With detectable PrEP drugs in dried blood spot (DBS) samples at the Week 26 visit |
| Key Secondary Outcomes | - Evaluate whether the intervention improves use of MOUD [Time Frame: 52 weeks] - Evaluate whether the intervention increases rates of viral suppression among people living with HIV [Time Frame: 52 weeks] - Evaluate whether the intervention increases use of PrEP among participants without HIV at enrollment [Time Frame: 26 weeks and 52 weeks] - Evaluate whether the intervention reduces opioid and polysubstance use at 26 and 52 weeks [Time Frame: 26 weeks and 52 weeks] - Evaluate whether the intervention reduces prevalence of bacterial STIs [Time Frame: 26 weeks and 52 weeks] - Evaluate whether the intervention reduces the rate of fatal overdose events by 26 and 52 weeks [Time Frame: 26 weeks and 52 weeks] - Evaluate whether the intervention reduces the rate of non-fatal overdose events by 26 and 52 weeks [Time Frame: 26 weeks and 52 weeks] - Assess whether the intervention increases the proportion of participants with undetectable HCV RNA among those with chronic HCV infection at enrollment [Time Frame: 26 weeks and 52 weeks] - Evaluate whether the intervention reduces HCV incidence [Time Frame: 52 weeks] - Evaluate whether the intervention increases rates of viral suppression among participants living with HIV at enrollment [Time Frame: 26 weeks] - Evaluate whether 26 weeks of "one stop" integrated health services delivered in a mobile health delivery unit, supported by peer navigation, increases MOUD use [Time Frame: 26 weeks and 52 weeks] - Evaluate whether 26 weeks of "one stop" integrated health services delivered in a mobile health delivery unit, supported by peer navigation, increases viral suppression at 26 and 52 weeks [Time Frame: 26 weeks and 52 weeks] - Evaluate whether 26 weeks of "one stop" integrated health services delivered in a mobile health delivery unit, supported by peer navigation, increases PrEP use at 26 and 52 weeks [Time Frame: 26 weeks and 52 weeks] - Evaluate whether 26 weeks of peer navigation to similar health services available at community-based agencies increases MOUD use at 26 and 52 weeks [Time Frame: 26 weeks and 52 weeks] - Evaluate whether 26 weeks of peer navigation to similar health services available at community-based agencies increases viral suppression at 26 and 52 weeks [Time Frame: 26 weeks and 52 weeks] - Evaluate whether 26 weeks of peer navigation to similar health services available at community-based agencies increases PrEP use at 26 and 52 weeks [Time Frame: 26 weeks and 52 weeks] - Assess the prevalence of SARS-CoV-2 seropositivity at baseline, 26 and 52 weeks [Time Frame: Baseline, 26 weeks, and 52 weeks] - Document the impact of the COVID-19 epidemic on participants' experiences of seeking, obtaining and/or maintaining health services, housing, food security and drugs [Time Frame: Up to 52 weeks] |
| Ethics Review | Status:  Approved  Date of approval:  Version 2.0 - March 27^th^ 2023  Name and contact details of Ethics committee(s):  Advarra IRB  6100 Merriweather Drive  Suite 600  Columbia, MD 21044  410.884.2900 |
| Completion date | 2024-09-30 (estimated) |
| Summary Results | Pending |
| IPD sharing statement | No |

**Supplemental Table 2. Footnotes for Table 3**

| 1 Between the Enrollment Visit and the 26-week visit, participants in the intervention arm will engage with study staff for clinical care in the mobile unit at a frequency determined by clinical need. These are considered care visits. Specimen collection and testing at care visits will be as needed for clinical care. Active control arm participants will not have these visits. |
| --- |
| 2 Assessment for COVID-19 will consist of a symptom screen and temperature. Details included in the SSP Manual. |
| 3 Assessment for OUD will be performed using a tool provided in Section 9 of the SSP Manual. Sites may assess for OUD at either the Screening or Enrollment Visit. If OUD is confirmed at screening, it does not need to be reassessed (confirmed) at enrollment. |
| 4 See SSP Section 4 for further guidance about eligibility assessment related to evidence of recent injection drug use and sharing of injection equipment. |
| 5 See SSP Section 4 for further guidance about eligibility assessment related to MOUD history and HIV risk behaviors. |
| 6 Targeted medical history to include participation in other interventional studies, overdose events, and follow-up of unresolved AEs/SAEs identified previously. |
| 7 Physical exam at enrollment to include vital signs, height, weight, general appearance, mouth and throat, neck, chest, abdomen, extremities and skin. Additional elements at clinician’s discretion for patient care. |
| 8 HIV treatment (or referral) will be offered at the first visit where HIV infection is confirmed, for those participants not already in treatment. Intervention arm participants will be offered HIV treatment in the mobile unit if the available regimen is appropriate for them. Intervention arm participants who require a different regimen and active control arm participants will be referred for treatment. Initiation of MOUD treatment will be the clinical priority, so people living with HIV may defer initiating HIV treatment until established on MOUD. |
| 9 The exact timing of COWS assessment and MOUD initiation will depend on clinician judgment, the readiness of the participant to begin treatment and other factors. |
| 10 Vaccination referral or treatment/treatment referral will be offered at the first visit where results from testing are available. |
| 11 For participants in the intervention arm receiving care in the mobile health delivery unit, ART regimens can be selected by study clinicians that treat HIV as well as HBV. Active control arm participants will receive referrals indicating their dual infection with HIV and HBV so that they may also receive appropriate treatment. |
| 12 STI results and referrals provided on a date after results are available, coded as a “split visit”. |
| 13 The types of samples collected (oropharyngeal, rectal, vaginal) are specified in the SSP Manual. |
| 14 See SSP Manual for instances when the HIV rapid test at Enrollment may be waived. |
| 15 HIV testing required for participants who were not previously confirmed to be living with HIV. |
| 16 Other HIV-related testing may be performed for clinical care. This may include HIV drug resistance testing and/or HLA-B5701 testing. If indicated, this testing should be performed at a local laboratory; these results will not be reported to the HPTN SDMC. |
| 17 Testing for medications used to treat substance use. |
| 18 Testing for substances of abuse. See SSP Manual Section 4 for guidance about eligibility related to detection of opioids in urine. |
| 19 Testing for pregnancy (urine human chorionic gonadotropin [HCG] testing) for any participant who could potentially be pregnant at that visit (unless already known to be pregnant). |
| 20 Perform HCV Ab testing at enrollment for all participants; perform HCV Ab testing at week 52 for participants who tested HCV negative at enrollment. |
| 21 Perform HCV viral load testing at enrollment, 26 weeks, and 52 weeks for participants who have a positive HCV Ab test. HCV RNA viral load testing may be performed on a date after HCV Ab results are available. |
| 22 Perform HBV viral load testing for participants with chronic HBV infection (HBsAg+) or isolated HBcAb positive for clinical care management (intervention arm only). |
| 23 The following tests are required: hemoglobin, creatinine, ALT, AST and total bilirubin. Sites may obtain these values by ordering a complete blood count and comprehensive metabolic panel if that is standard practice or less costly than performing individual tests. |
| 24 Plasma will be stored at Screening, Enrollment, 26 and 52 week visits, and at any visits where laboratory-based HIV testing is performed. Stored plasma will be used for testing at the HPTN LC. |
| 25 Stored urine will be used for testing at the HPTN LC. |
| 26 Stored DBS will be used for testing at the HPTN LC. |
| 27 Stored serum will be used for retrospective testing at the HPTN LC to determine the prevalence of SARS-CoV-2 seropositivity at baseline, 26 and 52 weeks; stored samples may also be used for specialized testing related to COVID-19. |
| Abbreviations: Ab: antibody; ART: antiretroviral treatment; aPTT: activated partial thromboplastin time; COWS: Clinical Opiate Withdrawal Scale; DBS: dried blood spot; GC/CT: gonorrhea/chlamydia; HAV: hepatitis A virus; HBV: hepatitis B virus; HBcAb: HBV core antibody; HBsAb: HBV surface antibody; HBsAg: hepatitis B surface antigen; HCV: hepatitis C virus; HLA: Human Leukocyte Antigen; MOUD: medications for opioid use disorder; NAAT: nucleic acid amplification test; OUD: opioid use disorder; PT: prothrombin time; SDMC: Statistical and Data Management Center; SSP: Study specific protocol; STI: sexually transmitted infection. |

# Supplemental Table 3. Additional procedures for participants who have a reactive or positive HIV test after enrollment

|  | Confirmatory visit |
| --- | --- |
| **Administrative and Behavioral Procedures** | |
| HIV counseling and test results | X |
| **Clinical Evaluations/Procedures** | |
| Initiate or refer for HIV treatment^1^ | X |
| Blood collection | X |
| **Laboratory Evaluations/Procedures** | |
| HIV testing (see SSP Manual)^2^ | X |
| HIV viral load | X |
| CD4 cell count | X |
| Other HIV related testing^3^ | X |
| Plasma storage^4^ | X |
| DBS storage^5^ | X |
| ^1^ For participants with documentation of confirmed HIV infection.  ^2^ Sites also ensure that local guidelines for HIV confirmatory testing are followed.  ^3^ Other HIV testing may be performed for clinical care. This may include HIV drug resistance testing and/or HLA- B5701 testing. If indicated, this testing should be performed at a local laboratory; these results will not be reported to the HPTN SDMC.  ^4^ Stored plasma will be used for testing at the HPTN LC.  ^5^ Stored DBS will be used for testing at the HPTN LC. | |
